# High‐throughput single‐cell DNA methylation and chromatin accessibility co‐profiling with SpliCOOL‐seq

**DOI:** 10.1002/ctm2.70584

**Published:** 2026-01-28

**Authors:** Qingmei Shen, Enze Deng, Ling Luo, Jingna Zhang, Qifeng Yang, Dan Su, Xiaoying Fan

**Affiliations:** ^1^ GMU‐GIBH Joint School of Life Sciences The Fifth Affiliated Hospital of Guangzhou Medical University, Guangzhou Medical University Guangzhou China; ^2^ Guangzhou National Laboratory Guangzhou International Bio Island Guangzhou China; ^3^ MOE Key Laboratory of Gene Function and Regulation Guangdong Province Key Laboratory of Pharmaceutical Functional Genes State Key Laboratory of Biocontrol School of Life Sciences Sun Yat‐sen University Guangzhou China

**Keywords:** cancer biomarkers, chromatin accessibility, DNA methylation, epigenetic ageing, single‐cell multi‐omics, SpliCOOL‐seq

## Abstract

**Background:**

DNA methylation and chromatin accessibility are pivotal epigenetic regulators of gene expression and cellular identity, with significant implications in tumorigenesis and progression. Current single‐cell multi‐omics methods are limited in throughput and sensitivity, hindering comprehensive biomarker discovery.

**Methods:**

We developed single‐cell split‐pool ligation‐based multi‐omics sequencing technology (SpliCOOL‐seq), a high‐throughput single‐cell sequencing technology that simultaneously profiles whole‐genome DNA methylation and chromatin accessibility in thousands of cells. By integrating in situ GpC methylation, universal Tn5 tagmentation, and split‐pool combinatorial barcoding, SpliCOOL‐seq achieves enhanced sensitivity and scalability.

**Results:**

SpliCOOL‐seq accurately distinguished lung cancer cell types based on genetic and multiple epigenetic modalities and revealed that the two DNA methyltransferase (DNMT) inhibitors, 5‐Azacitidine and Decitabine, both cause large‐scale demethylation but in distinct patterns. Applied to primary lung adenocarcinoma, SpliCOOL‐seq identified tumour subclones within the tumour lesion and uncovered novel DNA methylation biomarkers (e.g., *FAM124B, SFN, OR7E47P*) associated with patient survival. Additionally, we demonstrated accelerated epigenetic ageing and mitotic activity in tumour subclones, providing new insights into tumorigenesis.

**Conclusion:**

SpliCOOL‐seq achieves parallel profiling of whole‐genome DNA methylation and chromatin accessibility in the same individual cells in a high‐throughput manner and is hopefully used to illustrate regulatory interactions under different cell states. SpliCOOL‐seq enables high‐resolution, multi‐modal epigenetic profiling at single‐cell resolution, offering a powerful platform for discovering cancer biomarkers. Its application reveals novel therapeutic targets and early‐diagnostic markers, underscoring its potential in precision oncology.

**Key points:**

- SpliCOOL‐seq achieves high‐throughput single‐cell co‐profiling of DNA methylation and chromatin accessibility.
- DNMT inhibitors caused cancer cell demethylation with divergent patterns.
- SpliCOOL‐seq enables the discovery of genes related to LUAD tumorigenesis.
- Ageing and LUAD tumorigenesis may share similar epigenetic alterations.

## Graphical abstract

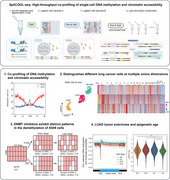

## 1 INTRODUCTION

Both chromatin accessibility and DNA methylation are critical regulators controlling cell‐type‐specific gene expression patterns. DNA methylation, predominantly occurring at CpG dinucleotides, mediates transcriptional silencing by hindering transcription factor binding or recruiting of methyl‐binding proteins.^1^, ^2^, ^3^ Chromatin accessibility, reflecting nucleosome positioning and the activity of regulatory elements, determines the spatial availability of DNA for transcriptional machinery and enhancer‐promoter interactions.^4^ The interplay between these two epigenetic modalities is critical for maintaining cellular homeostasis. For example, global DNA methylation erasure in primordial germ cells coincides with chromatin remodelling, resetting pluripotency.^5^ Additionally, hypomethylation of oncogenic promoters and hypermethylation of tumour suppressor genes are often coupled with chromatin compaction at regulatory regions.^6^, ^7^ Thus, simultaneous detection and integrative analysis of whole‐genome DNA methylation alongside chromatin accessibility can provide a comprehensive understanding of epigenetic coordination in health and disease, offering unprecedented opportunities for biomarker discovery and therapeutic intervention.^8^, ^9^, ^10^, ^11^, ^12^, ^13^, ^14^

The scCOOL‐seq^8^ enables concurrent measurement of multiple epigenetic modalities within a single cell, leading to subsequent development of related multi‐omics methodologies, including scNOME‐seq,^9^ scNMT‐seq,^10^ iscCOOL‐seq,^11^ scChaRM‐seq,^12^ snmCAT‐seq,^13^ and scNOMeRe‐seq.^14^ However, the utilization of these multi‐omics techniques has been restricted to single‐cell lysates, which severely limits the cell throughput and incurs substantial costs and time consumption. Achieving high‐throughput single‐cell sequencing that can simultaneously detect genome‐wide DNA methylation and chromatin accessibility remains a challenge. Recently, sciMETv3^15^ supported the co‐profiling of DNA methylation and chromatin accessibility by employing another round of indexed Tn5 tagmentation before the intermediate nucleosome disruption step. Similar to sciMETv2,^16^ sciMETv3 conducted the initial round of cell labelling using Tn5 transposase labelled with different indexes, which would lead to variations for fragmentation among individual cells. Second, as the open chromatin regions constitute only a small portion of the genome, when co‐profiling by different Tn5 tags, very limited data could be allocated to the ATAC module for each cell, which reduces the detection sensitivity to open chromatin regions.

In this study, we introduce SpliCOOL‐seq, an improved method that combines split‐pool ligation‐based single‐cell indexing following in situ GpC methylation and tagmentation using universal Tn5 transposase. This approach enhances high‐throughput analysis of nucleosome‐depleted regions (NDRs), also known as chromatin‐accessible regions, alongside genome‐wide DNA methylation with improved data quality. SpliCOOL‐seq enables the identification of cell‐type‐specific epigenetic signatures and novel biomarkers in cancer. Applied to lung adenocarcinoma, SpliCOOL‐seq uncovered subclonal heterogeneity, candidate diagnostic markers, and accelerated epigenetic ageing, demonstrating its utility in translational cancer research. Overall, SpliCOOL‐seq represents a significant advancement in the repertoire of multimodal single‐cell profiling technologies, proving to be a valuable tool for tumour studies.

## 2 METHODS

### 2.1 Cell culture and cell nuclei isolation

Cell lines (GM12878, NIH/3T3, A549, NCI‐H460, SK‐MES) were cultured in 5% CO_2_ at 37°C. GM12878 and NCI‐H460 cells were grown in RPMI1640 (Gibco, C11875500CP) supplemented with 10% (v/v) FBP (HyCyte, C520), 1× penicillin‐streptomycin (Gibco, 15140122). NIH/3T3 and SK‐MES cells were grown in DMEM (Gibco, C11995500BT), with the same supplements as GM12878 cells. A549 cells were grown in Ham's F‐12K (Gibco, 21127022), with the same supplements as GM12878 cells. For cultured cells, they were washed with ice‐cold 1×PBS buffer (CELLCOOK, CM2018) and spun down at 4°C and 300*g* for 5 min. The cell pellet was then re‐suspended in ice‐cold NIB‐HEPES buffer (10 mM HEPES (Sigma‐Aldrich, H3375), 10 mM NaCl, 3 mM MgCl2, 0.1% Igepal (Sigma‐Aldrich, I8896), 1×cOmplete protease inhibitor cocktail, EDTA‐free (Roche, 11873580001) and 0.1% Tween (Sigma‐Aldrich, P9416)) and incubated on ice for 10 min. A repeated spin‐down step and resuspension in ice‐cold NIB‐HEPES buffer for a second incubation were performed. For clinical tissues, cell nuclei suspensions were prepared by douncing of the liquid nitrogen snap‐frozen tissues with NIB‐tris Buffer (10 mM Tris‐HCl, pH 7.5 (Sangon Biotech, B548139‐0500), 10 mM KCl, 3 mM MgCl2, 1×cOmplete protease inhibitor cocktail, EDTA‐free (Roche, 11873580001), 0.1% Tween (Sigma‐Aldrich, P9416) and 0.1% Igepal (Sigma‐Aldrich, I8896)). The nuclei were then washed with ice‐cold 1%BSA (Sigma‐Aldrich, V900933) after passing through a 40 µm cell strainer.

### 2.2 In vitro GpC methylation and nucleosome depletion

The single nuclei were washed with 1×DPBS buffer (CORNING, 21‐031‐CV) and subsequently incubated at 37°C for 30 min in a reaction mixture containing 1×GpC buffer (New England BioLabs, B0227), 0.6 U/µL M.CviPI methyltransferase (New England BioLabs, M0227L) and 0.6 mM/µL SAM (New England BioLabs, B9003). Then they were washed with ice‐cold NIB‐HEPES buffer and resuspended in 10 mL of NIB‐HEPES buffer containing 406 µL of 37% formaldehyde (Sigma‐Aldrich, F8775), making the final concentration of formaldehyde at 1.5% (the optimal concentration), and incubated at room temperature for 10 min. The fixation was terminated with 0.2 M glycine (Sigma‐Aldrich, 50046) and incubated on ice for 5 min. After centrifuging at 4°C and 500*g* for 5 min, the nuclei were washed with 1× NEB buffer 2.1 (New England BioLabs, B7202). Subsequently, the nuclei were resuspended in 800 µL of 1× NEB buffer 2.1, followed by the addition of 12 µL 20% SDS (final con. 0.3%) and incubated at 60°C for 10 min for nucleosome depletion.^17^ 200 µL of 10% Triton‐X was added and incubated at 37°C for 30 min. The single nuclei were washed once with NIB‐tris Buffer and then resuspended in 100 µL of NIB‐tris Buffer.

### 2.3 Tagmentation and barcoding

To generate the universal adaptor embedded Tn5 transposase complex, 20 µM I7 adaptor and 20 µM anneal primer (Table S7) were subjected to annealing in a thermocycler using the following program: 75 °C for 15 min, 60 °C for 10 min, 50 °C for 10 min, 40°C for 10 min, and 25°C for 30 min. Subsequently, 9 µL of the annealed 10 µM universal I7 adaptor was combined with 10 µL of unloaded Tn5 enzyme (Vazyme, S601‐01) and 17 µL of coupling buffer (Vazyme, S601‐01). The reaction mixture was gently mixed by pipetting up and down 20 times, followed by incubation at 30°C for 1 h. The universal Tn5 transposase complex was then stored at –30 °C to –15 °C until further use. The nuclei were distributed into PCR tubes (20 000 nuclei per tube) that contained 2 µL Tn5 transposase complex, 4 µL 5×TTBL tagmentation buffer (Vazyme, S601‐01) and NF‐water up to a total of 20 µL. The mixture was incubated at 55°C for 30 min with rotation. Then all tagmented nuclei were pooled together and washed with 1 mL of 1×NEB buffer 3.1 (New England BioLabs, B7203). After centrifuging at 4°C and 1000*g* for 5 min, the nuclei were resuspended in 478 µL of 1× NEB buffer 3.1, followed by the addition of 98.4 µL of 10×T4 DNA Ligase Buffer and 24 µL of T4 DNA Ligase (ABclonal, RK21500). Next, 12 µL ligation mix was distributed to Barcode Plate 01, which contained 8 µL of 5 µM pre‐annealed barcode A primer (Table S7) and incubated at room temperature for 30 min with rotation. After the first round of ligation, 8 µL of 10 µM Blocking01‐Solution (50 µL of 100 µM Blocker01 oligo, 50 µL of 10× T4 DNA Ligase Buffer and 400 µL of nuclease‐free water) was then added to each well, and the blocking reaction was continued at room temperature for 30 min with rotation. The nuclei were pooled together and washed with 1 mL of 1×NEB buffer 3.1. Following centrifuging at 4°C and 1000*g* for 5 min, the nuclei were resuspended in 956 µL of 1× NEB buffer 3.1, supplemented with the addition of 196.8 µL of 10×T4 DNA Ligase Buffer and 48 µL of T4 DNA Ligase. Subsequently, 12 µL ligation mix was dispensed into Barcode Plate02 along with an additional volume of 8 µL 5 µM pre‐annealed barcode B primer. The ligation was performed by incubating the mixture at room temperature for 30 min with rotation. After the second round of ligation, 8 µL of 10 µM Blocking02‐Solution (100 µL of 100 µM Blocker02 oligo, 100 µL of 10× T4 DNA Ligase Buffer and 800 µl of nuclease‐free water) was added to each well, and the reaction was continued at room temperature for 30 min with rotation.

### 2.4 Bisulfite conversion and library preparation

The barcode‐ligated nuclei were divided into 200 nuclei per tube and treated with proteinase K (Roche, P8107S), followed by conversion using a Hieff Superfast DNA Methylation Bisulfite Kit (YESEN, 12225ES50) in accordance with the provided instructions. Each tube was eluted in 20 µL elution buffer and transferred to PCR tubes containing the following reaction mixture for random priming and extension: 2.5 µL of 10×blue buffer (TIANGEN, NG202), 1 µL of 10 mM dNTP mixture (TaKaRa, 4019), and 1 µL of 10 µM random primer (Table S7). The reactions were heat‐shocked at 95°C for 45 s and then rapidly cooled on ice for 2 min. Subsequently, each reaction was supplemented with 25 U Klenow (3′→5′exo‐) polymerase (TIANGEN, NG202), and incubated at a slow ramp +1°C/15 s up to 37°C for 60 min. After random priming and extension, the products were purified with 1× AMPure XP Beads (Beckman, A63882) and eluted with 21 µL of elution buffer. Then they were transferred to a PCR tube containing 25 µL of 2× KAPA HiFi HotStart ReadyMix (KAPA BioSystems, KK2602), along with 2 µL of 10 µM QP2 primer and 2 µL of 10 µM N5XX index primer (AATGATACGGCGACCACCGAGATCTACACNNNNNNNNTCGTCGGCAGCGTCAGATGT), following the PCR program: 98°C for 3 min, 98°C for 20 s, 65°C for 30 s, 72°C for 30 s, step2 to 4 with 12cycles, 72°C for 5 min and 4°C hold. The libraries were purified with 0.8× AMPure XP Beads and eluted with 20 µL of elution buffer.

### 2.5 Library quantification and sequencing

The libraries were quantified using the Qsep100 system to evaluate the size distribution of DNA fragments, while the concentration was determined using the Qubit 4.0 fluorometer. Subsequently, these libraries were sequenced on the SURFSeq 5000 sequencer in a 150 PE run mode.

### 2.6 scATAC‐seq library preparation and sequencing

The three lung cancer cell lines were combined in equal proportions to create a single‐nucleus suspension following the protocol outlined in the Chromium Next GEM Single Cell ATAC Reagent Kits v2 (10×Genomics, PN‐1000406) User Guide for cell capture and library preparation. The library was subjected to quality control using the Qsep100 system and quantified with the Qubit 4.0 fluorometer. Subsequently, sequencing was performed on the SURFSeq 5000 platform using a 150 PE run mode.

### 2.7 Lentivirus production and generation of stable knockout cell lines

To generate *SFN*‐knockout and control cell lines, sgRNAs targeting human *SFN* or a non‐targeting control sequence were cloned into the BsmBI site of the pRlenti‐Cas9‐Puro vector (Addgene), yielding pRlenti‐sgSFN‐Cas9‐Puro and pRlenti‐sgNT‐Cas9‐Puro, respectively. HEK293T cells were co‐transfected with each lentiviral construct along with the packaging plasmids psPAX2 and pMD2.G using polyethylenimine. Viral supernatants were harvested 48 h post‐transfection, filtered through a.45 µm PVDF membrane, and used immediately for infection. A549 and H460 cells were infected in the presence of 6 µg/mL polybrene and subjected to spinoculation (800*g*, 45 min, 4°C). 12 h after infection, the medium was replaced with fresh complete medium. Selection was initiated 48 h post‐infection using 1 µg/mL puromycin and maintained for 6 days, with medium replenishment every 2 days. The resulting polyclonal populations of puromycin‐resistant cells were utilized in subsequent experiments.

### 2.8 Cell proliferation assay

Cell proliferation was assessed using the cell counting kit‐8 (CCK‐8) assay. The established *SFN*‐knockout (sg*SFN*) and control (sgNT) cells were harvested by trypsinization with.05% trypsin, counted, and seeded into 96‐well plates at a density of 400 cells per well in 100 µL of complete culture medium. Cell proliferation was monitored continuously starting 24 h after seeding (Day 1 baseline), with absorbance measurements recorded at 450 nm at 2‐day intervals over a four‐timepoint period. For each measurement, 10 µL of the CCK‐8 reagent was added directly to each well, followed by incubation for 1 h at 37°C before reading the optical density. The medium containing CCK‐8 was refreshed before each measurement to ensure a continuous nutrient supply. All experiments were performed with at least six replicate wells per group and repeated independently three times.

### 2.9 Apoptosis assay by flow cytometry

Apoptosis was quantitatively assessed using an Annexin V‐FITC/PI apoptosis detection kit. Briefly, the established sg*SFN* and sgNT cells were trypsinized with 0.05% trypsin, counted, and seeded into 12‐well plates at an appropriate density to reach approximately 70%–80% confluence on the day of analysis. Each experimental group was set up in triplicate wells. After 72 h of culture under standard conditions, both adherent and floating cells were collected. Cells were processed as described in the manufacturer's instructions for the kit. The stained cells were analyzed immediately using a flow cytometer (Beckman CytoFLEX S). Data analysis was performed using flow cytometry analysis software (FlowJo), and cells were categorized as viable (Annexin V^−^/PI^−^), early apoptotic (Annexin V^+^/PI^−^), late apoptotic (Annexin V^+^/PI^+^), or necrotic (Annexin V^−^/PI^+^). The experiment was independently repeated at least three times.

### 2.10 Cell migration assay (Transwell)

Cell migration was evaluated using a Transwell chamber assay (8 µm pore size). The constructed sg*SFN* and sgNT A549 cells were harvested by trypsinization with 0.05% trypsin, counted, and resuspended in complete medium. In a 200 µL complete culture medium suspension, 6 × 10^4^ cells were inoculated into the upper chamber of each insert. After 12 h, switch to a low‐serum medium with 1% FBS. The lower chamber of a 24‐well plate was filled with 600 µL of medium supplemented with 10% FBS as the induction condition. The cells migrated for 12 h in a humidified incubator at 37°C and 5% CO_2_. After incubation, non‐migratory cells on the upper surface of the membrane were carefully removed with a cotton swab. Cells that had migrated to the lower surface were fixed with 4% paraformaldehyde for 20 min, stained with.1% crystal violet solution for 15 min, and rinsed gently with PBS. Photographed under an inverted light microscope using consistent magnification for all samples (10×). The number or area of migrated cells was quantified by analyzing the captured images with ImageJ software (National Institutes of Health). For each experimental group, three independent replicates were performed, and the assay was repeated at least three times.

### 2.11 Pre‐processing of SpliCOOL‐seq data

The two linker sequences were precisely identified at the fixed position within read 1. Reads lacking these linker sequences were classified as unknown structure. The original barcodes were extracted from the linker sequence and compared against those in the whitelist by calculating the minimum Levenshtein distance. Barcode with a minimum distance exceeding 2 was labelled as an unknown barcode and discarded. Trimming and low‐quality read filtering were performed using Trim Galore (version 0.6.10, https://github.com/FelixKrueger/TrimGalore) with parameters –length 30 and –quality 20. Reads were then mapped to the mouse GRCm39 or human GRCh38 reference genome using Bismark (version 0.24.2)^18^ with Bowtie2 (version 2.5.2),^19^ applying paired‐end and non‐directional mapping. After paired‐end alignment, unmapped reads were realigned in single‐end mode to the same reference genome in single‐end mode. PCR duplicates were identified and removed based on their genomic coordinates using the Picard MarkDuplicates tool (https://broadinstitute.github.io/picard/, version 3.1.1), with the command parameter: “BARCODE_TAG = CB”. Only non‐duplicated reads were retained for downstream analysis.

### 2.12 Quantification of WCG and GCH methylation levels

The methylation levels of cytosines classified as WCGs (W = A or T) and GCHs (H = A, T, or C) with a minimum coverage of one read (≥1× depth) were quantified using methylpy (version 1.4.7)^20^ with the call‐methylation‐state command. For each WCG or GCH site, the methylation level was calculated as the ratio of reads supporting cytosine (C) to the total number of reads covering the site. The average DNA methylation level and chromatin accessibility for all cells were derived from the mean methylation levels of WCG and GCH sites, respectively. Bisulfite conversion efficiency was assessed by examining the WCH methylation level in mitochondrial DNA. Bigwig files were generated using bedGraphToBigWig (version 2.10).^21^

### 2.13 Defining NDRs

We identified single‐cell NDRs in individual cells by adapting methodologies from a prior study,^22^ employing a custom Python script (from https://github.com/sherryxue‐PKU/scNanoCOOL‐seq). The detection process can be distilled into three key steps. First, the bam files of a certain group of single cells (either a cell type or a treatment condition) were merged as a pseudo‐bulk sample. Then the methylation information at the GCH sites was extracted using the call‐methylation‐state command from the methylpy software.^20^ A sliding‐window strategy (with a window length of 100 bp and a step length of 20 bp) was then applied to partition the methylation data of GCH sites into numerous tiles. For each tile, the total number of methylated sites (Cs) and the total number of covered GCH sites (Cs + Ts) were compared with the corresponding chromosomal background using the scipy.stats.chi2 function from the SciPy Python library.^23^ Tiles meeting the criteria of a *p*‐value less than 1e‐10, coverage of at least one read, and containing more than five GCH sites were designated as NDRs for downstream analysis. Finally, BEDTools (version 2.31.1)^24^ was utilized to merge overlapping NDRs, yielding cell‐type‐specific NDRs.

### 2.14 Processing of RNA‐seq data from ENCODE

Raw counts data were obtained from the ENCODE database (ENCFF876SRX, ENCFF147QDQ, ENCFF799PDP, ENCFF328YIW, ENCFF024DUU, ENCFF697YYW, ENCFF477TRE) and the Gene Expression Omnibus (GEO) repository (GSE95536). DEG analysis was conducted using the DESeq function from the DESeq2 (version 1.42.1) package,^25^ with significance defined by an adjusted *p*‐value threshold of less than.05.

### 2.15 Cell clustering with WCG, GCH and NDR

The WCG and GCH methylation calls were utilized to construct a matrix of cells × 250 kp genomic window. Cells with fewer than 10 000 bins covered and bins with less than 50% of cell coverage were excluded. Missing values in the matrix were imputed using the mean methylation level of each cell. Principal component analysis (PCA) was performed on the matrix to reduce noise, retaining the top 50 principal components for further analysis. A neighbourhood graph was constructed to estimate the relationships between data points, and the Leiden algorithm was employed to detect communities and identify cell clusters.^26^ Additionally, the same principal components were used for non‐linear dimensionality reduction, generating a Uniform Manifold Approximation and Projection (UMAP) visualization.

### 2.16 scATAC‐seq data analysis

The 10x Genomics Cell Ranger‐ATAC (version 2.1.0) pipeline was executed on the raw sequencing data to generate a count matrix for peaks and transcription factors. The obtained fragments file was loaded, and the snapatac2 (version 2.7.0)^27^ workflow was used for downstream analyses. Cell quality was evaluated based on a minimum TSS enrichment score of 10 and a fragment count range of 5000 to 100 000 to filter cells. Feature selection was conducted using the snap.pp.select_features function with the parameter n_features = 250 000. Potential doublet cells were identified and removed by calculating the doublet score for each cell using snap.pp.scrublet. Following quality control, a total of 5662 cells were retained for further analysis. Spectral embedding was applied for dimensionality reduction, and UMAP was utilized to project the cells into a two‐dimensional space for visualization. Graph‐based clustering was performed by constructing a k‐nearest neighbour graph using snap.pp.knn, followed by the application of the Leiden community detection algorithm to identify densely connected clusters. Gene activity matrices were computed for each cell using the snap.pp.make_gene_matrix function, and cell type‐specific peaks were called using MACS3 (version 3.0.2)^28^ via the snap.tl.macs3 function.

### 2.17 Single‐cell CNV analysis

The CNVs inferred from the SpliCOOL‐seq data were primarily derived from read counts across fixed‐length windows using Control‐FREEC(version 11.6).^29^ The parameters for window size and ploidy were set to 1 000 000 and 2, respectively, in the configuration file. Cells were aggregated into pseudo‐bulk profiles by cell type to compute cell type‐specific CNVs. The coefficient of variation (CV) for individual cells was determined based on the inferred copy number ratios. Public whole‐genome sequencing (WGS) data were acquired from the ENCODE database (ENCFF022XPK, ENCFF122NPY, ENCFF534EUU, and ENCFF846WHK).

### 2.18 DMR and DMS analysis

Differentially methylated regions (DMRs) were identified using MethScan (version 1.1.0) software^30^ with the methscan diff function, employing a bandwidth parameter of 100. Differentially methylated sites were identified using the parameters –stepsize 1 and –bandwidth 1. The identified DMRs were subsequently merged using BEDTools (version 2.31.1)^24^ and clustered via ClusterGvis (https://github.com/junjunlab/ClusterGVis, v0.1.2)

### 2.19 Motif analysis

For each cell type or clone, de novo motif discovery was performed within differentially methylated regions using the HOMER (version 4.11)^31^ suite via the findMotifsGenome.pl command, with the parameters ‐mask and ‐size 200. Parallel analyses were conducted for NDRs in each cell type, as well as for differentially accessible regions.

### 2.20 Epigenetic ageing analysis

To investigate methylation age in cancer cells, we utilized the scAge (version 1.0.0)^32^ framework to estimate the epigenetic age at the single‐cell level. The model was trained using 450k methylation array data from lung adenocarcinoma (LUAD) samples sourced from TCGA, with downsampling performed using CpG sites overlapping with single‐cell data for a fivefold across‐validation. Methylation values were binarized to quantify the methylation frequency of WCG sites in each cell. Undetected CpG sites in single‐cell data were imputed using CpG Transformer.^33^ Statistical concordance (*p* < .05, hypergeometric test) was established between age‐associated CG sites and epigenetic clock datasets.

### 2.21 Enrichment for gene set annotations

Analysis of gene set enrichment was performed using clusterProfiler^34^ to elucidate the characteristics of multiomics data. Functional enrichment analysis of peaks was conducted employing Cistrome‐GO.^35^ Bar graph of enriched terms were utilized the web‐based platform Metascape (https://metascape.org/gp/index.html#/main/step1).

### 2.22 Survival analysis

We identified a range of differentially methylated promoter genes and performed comprehensive overall survival analysis for each utilizing the web‐based platform Kaplan–Meier survival analysis (https://kmplot.com/analysis/).

### 2.23 RNA‐seq analysis

To identify differentially expressed genes between sample groups sg*SFN* and sgNT, raw sequencing reads were subjected to quality control using fastp (version 0.23.4)^36^ to remove low‐quality sequences. Processed reads were aligned to the Homo sapiens reference genome GRCh38 (ENSEMBL release 111) using STAR (v2.7.11b).^37^ Differential expression analysis comparing sg*SFN* samples against sgNT controls was executed using DESeq2 (v1.42.1)^25^ with default parameters. A significance threshold of adjusted *p*‐value < .05 (Benjamini‐Hochberg correction) and absolute log_2_ fold change >1 was applied to classify DEGs.

## 3 RESULTS

### 3.1 SpliCOOL‐seq enables co‐profiling of DNA methylation and NDRs in a high‐throughput manner

**FIGURE 1:**
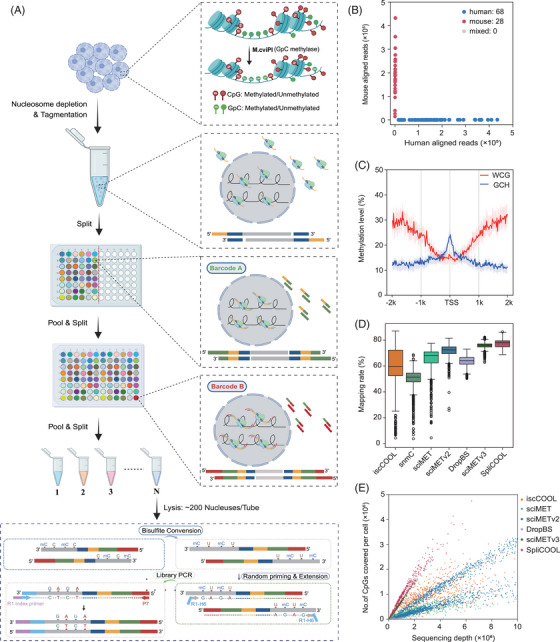
SpliCOOL‐seq overview and performance. (A) Schematic of SpliCOOL‐seq. Single nuclei were treated with M.CviPI in situ, followed by fixation and disruption of the nucleosomes. Then, nuclei were barcoded through split‐pool combinatorial indexing after universal Tn5 tagmentation. The nuclei were subsequently lysed, followed by bisulfite conversion, random priming and extension. The final library was obtained through PCR amplification. (B) Scatter plot illustrating the number of reads mapped to the human and mouse genomes in each cell from the species‐mixing experiment. (C) WCG and GCH methylation levels of GM12878 cells within ± 2 kb of the transcription start site (TSS) as determined by SpliCOOL‐seq, with shaded regions indicating the 25th and 75th percentiles of methylation levels across cells. Cell number *n* = 84. (D) Comparison of mapping efficiencies across individual cells by each method. The mapping rate was calculated by comparing the number of aligned reads to that of adaptor‐trimmed reads in each sample. (E) Scatter plot depicting the number of CG dinucleotides covered by the total aligned reads per cell by each method.

We presented a strategy that enables the concurrent assessment of genome‐wide DNA methylation (WCG methylation) and chromatin accessibility (NDRs, via GCH methylation) in individual cells (Figure 1A). Initially, in situ methylation of GpC sites was performed while maintaining the chromatin structure. The fixation of cell nuclei is critical and should be occurred after GpC methylation treatment using an appropriate concentration of formaldehyde (Figure S1A–C). Unlike previous single‐molecule combinatorial indexing(sci)‐based methods,^16^, ^38^, ^39^ we utilized universal Tn5 transposase complexes. This approach was adopted to avoid variations in Tn5 transposase activity and fragmentation efficiency that may arise from incorporating Tn5 transposase with different barcodes within each reaction chamber. Furthermore, competition between Tn5 transposons with distinct barcodes during amplification leads to uneven genomic coverage across cells.^40^ As a result, we achieved more uniform data quantity across cells compared with sciMETv2^16^ (Figure S1D, Table S1). Published single‐cell whole genome bisulfite sequencing methods utilizing post‐bisulfite adapter‐tagging (PBAT) often exhibit suboptimal mapping efficiency,^41^ likely due to substantial generation of chimera fragments with multiple rounds of random priming.^40^ The sciMET^39^ study has demonstrated that increasing the number of random priming rounds (four rounds versus three rounds) can improve library coverage, but this benefit was observed only under conditions of deep sequencing (a total of 1 × 10^7^ aligned reads per cell). Such a high sequencing depth is not currently feasible for high‐throughput single‐cell methylation sequencing. We observed larger library sizes, and lower mapping ratios when conducting four rounds of random priming and extension, while the reads duplication rate was significantly lower (Figure S1G–I). Consequently, both processes showed comparable effects on genome coverage under limited sequencing depth (Figure S1J). The implementation of fluorescence activated cell sorting (FACS) in the final splitting step has the potential to reduce sequencing background, but no significant difference was observed in the read ratio within cells (∼90%) (Figure S1H,I).

Species‐mixing experiments revealed a negative correlation between the number of cells per tube in the final splitting step and the anticipated collision rate. We allocated 20 000 cells for barcode ligation, achieved a recovery rate of approximately 25%, and subsequently partitioned the recovered cells into groups of 100, 200, and 300 cells for library preparation and sequencing. When the cell count per tube was 100, doublets were virtually absent, while the expected collision rate was approximately 13.5% for barcodes with 300 cells per tube (Figure 1B; Figure S1B). Consistent with previous studies,^9^, ^10^ SpliCOOL‐seq detected gene transcription start sites (TSS) with DNA hypomethylation and enhanced accessibility (Figure 1C). In comparison to earlier single‐cell DNA methylation sequencing techniques,^11^, ^15^, ^16^, ^39^, ^42^, ^43^ SpliCOOL‐seq achieved a superior read mapping rate (from 69.3% to 87.6%) and detected a greater number of CpG sites at equivalent sequencing depths within individual cells (Figure 1D,E). As our data exhibited stronger enrichment in CpG‐dense regions (Figure S1J). Notably, at the resolution of single WCG sites, approximately 100 cells can achieve 80% (≥1× depth) and 70% (≥5× depth) genomic coverage, respectively (Figure S1K). Collectively, our findings demonstrate that SpliCOOL‐seq proficiently captures both DNA methylation status and chromatin accessibility at single‐cell level in a high‐throughput manner.

### 3.2 SpliCOOL‐seq clearly distinguishes different lung cancer cells at multiple omics dimensions

**FIGURE 2:**
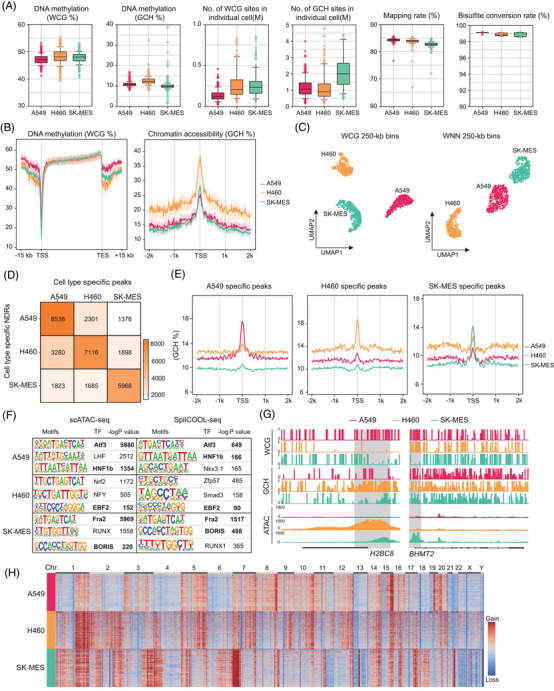
SpliCOOL‐seq distinguishes different cell types based on multiple modalities. (A) Global DNA methylation levels (as measured by the modification levels of WCG%), chromatin accessibility (as measured by the modification levels of GCH%), the numbers of WCG sites and GCH sites covered, mapping ratio, and bisulfite conversion rate estimated in individual cells of A549 cells, H460 cells and SK‐MES cells profiled by SpliCOOL‐seq. (B) DNA methylation levels within and surrounding gene bodies (left panel) and chromatin accessibility around the TSS (right panel) in single cells belonging to the three cell types. Solid lines represent the mean levels of single cells, while shaded areas indicate the 25th and 75th percentiles among the cells. (C) UMAP embedding showing cells based on their DNA methylation levels (WCG in 250‐kb non‐overlapping bins) and an integrated analysis of DNA methylation and chromatin accessibility (WNN in 250‐kb non‐overlapping bins). (D) Heatmap showing the intersection of cell‐type‐specific peaks identified through scATAC‐seq and NDRs detected via SpliCOOL‐seq. The corresponding numbers of overlapped regions are displayed within the boxes. (E) Chromatin accessibility around the TSS, which were cell type specific based on scATAC‐seq analysis. (F) Top enriched motifs for cell‐type‐specific accessible regions and NDRs are identified from different methods. *p*‐values are calculated using one‐sided Fisher's exact test. (G) Browser track showing the DNA methylation (WCG) and chromatin accessibility (GCH or ATAC) profiles at the *H2BC8* locus and *BHMT2* locus across various cell types. Promoter regions are highlighted by light grey shading. (H) Heat map showing the CNVs in individual A549, H460 and SK‐MES cells at 10 Mb resolution, as inferred from SpliCOOL‐seq data.

To evaluate the capability of SpliCOOL‐seq in resolving cell‐type‐specific DNA methylation and chromatin accessibility, we applied the technique to three lung cancer cell lines including A549, H460 and SK‐MES. A total of 1310 cells were obtained, yielding an average of 463 522 unique reads, 193 554 WCG sites (W includes A or T) and 1 689 066 GCH sites (H includes A, C or T) per cell (Figure 2A). The bisulfite conversion rate in all cell types exceeded 99% based on examining the methylation status of mitochondrial sequences^44^ (Figure 2A). We assessed the DNA methylation levels (WCG%) within gene regions and chromatin accessibility (GCH%) around the transcription start sites (TSS) for each cancer cell type (Figure 2B). All cells exhibited dropped WCG methylation levels at the TSS, while the gene body showed the highest WCG methylation compared with flanking regions. Although A549 cells displayed lower genome‐wide WCG methylation levels, they exhibited significantly higher methylation around gene‐franking regions (Figure 2A,B), indicating the importance of evaluating DNA methylation levels in distinct genome contexts. Furthermore, lower levels of DNA methylation did not correspond to higher chromatin accessibility in different cell types (Figure 2B), underscoring the complexity of epigenetic co‐regulation.

By partitioning the genome into 250‐kb bins, both WCG and GCH methylation data distinctly segregated the three cell types into separate clusters through unsupervised clustering (Figure 2C; Figure S2A). Integration of the two modalities using weighted‐nearest neighbor (WNN)^45^ yielded comparable clustering outcomes (Figure 2C). At the current sample size, DNA methylation was more effective at distinguishing different cancer cell types, with TSS methylation data alone sufficient to separate the cells in the UMAP analysis (Figure 2C; Figure S2B). To confirm that the NDRs we profiled represent true accessible chromatin regions, we also performed scATAC‐seq analysis on the same cells (Figure S2C,D). The cell type‐specific NDRs and cell type‐specific differentially accessible regions (DARs) showed substantial overlap (Figure 2D; Table S2). The DARs for each cell type showed higher GCH methylation levels in the corresponding cell type, and that NDRs for each cell type also showed higher ATAC signal in the corresponding cell type (Figure 2E; Figure S2E). Notably, the enriched motifs identified in both SpliCOOL‐seq and scATAC‐seq were identical in each cell type (Figure 2F), suggesting that SpliCOOL‐seq accurately captures open chromatin regions.

We then calculated the differential expressed genes (DEGs) across the cell types and found cell‐type specific DEGs (Table S2). For instance, we observed elevated GCH methylation levels of *H2BC8* and *H2AC8* in H460, which corresponded to peak‐rich regions in the scATAC‐seq analysis, while the WCG methylation levels at TSS of these genes remained significantly lower (Figure 2G; Figure S2F–H). Similarly, betaine‐homocysteine S‐methyltransferase2 (*BHMT2*), a marker for lung squamous cell carcinoma (LUSC) cell line SK‐MES,^46^ exhibited higher GCH methylation levels and lower WCG methylation levels in SK‐MES cells, indicating a negative regulatory relationship between the two modality at gene regions. We further categorized the genes into four groups based on their expression levels within each cell type (using ENCODE data; see methods). The promoter methylation levels gradually decreased while promoter accessibility increased in tandem with elevated gene expression (Figure S2I), consistent with observations from the previous study.^47^ Thus, SpliCOOL‐seq enables integrated analysis of DNA methylation and chromatin accessibility within the same single cells.

Since scCOOL‐seq also facilitates the analysis of copy number variations (CNVs), we assessed the ability of SpliCOOL‐seq to capture CNVs in different cancer cells. Although we observed reduced genome coverage compared with traditional scCOOL‐seq, SpliCOOL‐seq accurately captured the CNVs in each cancer cell types, allowing differentiation of various tumour clones (Figure 2H; Figure S2J), highlighting the superiority of SpliCOOL‐seq in capturing genomic features.

### 3.3 DNMT inhibitors exhibit distinct patterns in the demethylation of tumour cells

**FIGURE 3:**
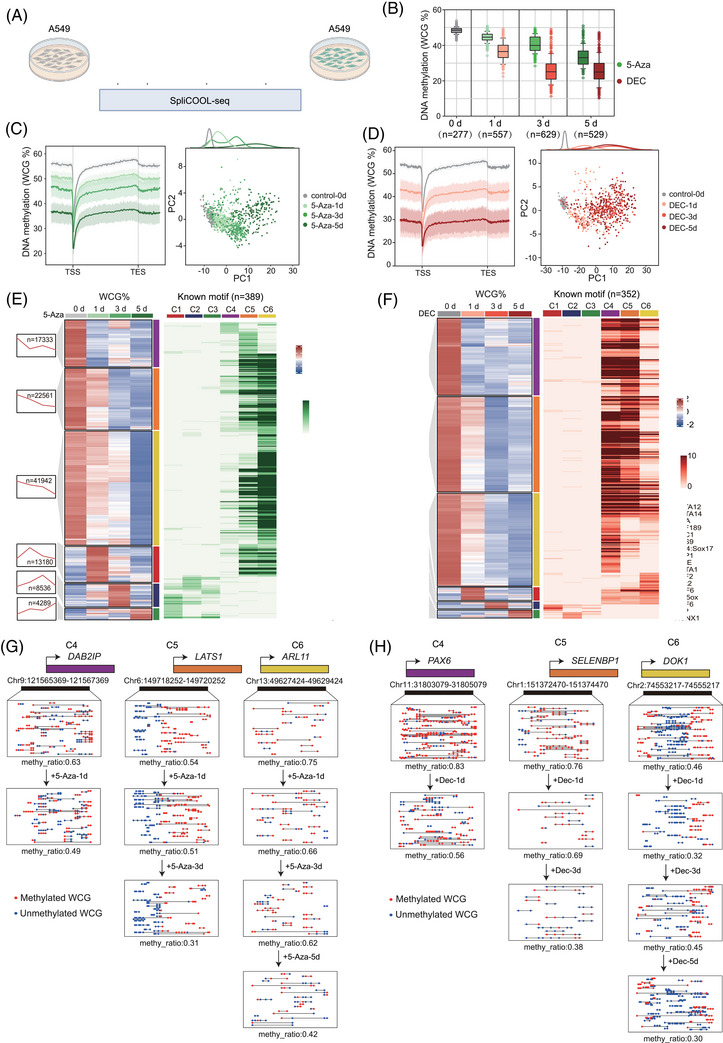
SpliCOOL‐seq detected distinct demethylation patterns of tumour cells under treatment with different DNMT inhibitors. (A) Schematic illustration of the treatment protocol for A549 cells with 5‐Aza or DEC. Cells are subjected to 1 µM concentrations of 5‐Aza or DEC for durations of 0, 1, 3 and 5 days. (B) Boxplot showing global WCG methylation levels in cells treated with 5‐Aza (green) or DEC (red) at each timepoint. (C, D) Average WCG methylation levels across the gene body in single cells after being treated with 5‐Aza (C) and DEC (D) for 0, 1, 3 and 5 days. 15 kb upstream of the TSS and 15 kb downstream of the TES were calculated (Left). The shading areas display the 25th and 75th percentiles of DNA methylation levels across cells. The right panels display Principal component analysis (PCA) of all the cells based on WCG in 250‐kb non‐overlapping bins. (E, F) Heatmaps showing the DMR clusters showing different methylating variations over time. The six clusters are represented in distinct colours on the right side of the heatmap (left). The enriched motifs in each cluster are shown in the right panels, with the TF lists of C1–C3 clusters presented. (G, H) The three clusters of DNA demethylation patterns of TSGs in response to the 5‐Aza treatment (G) or DEC (H) treatment. Red circles and blue circles represent methylated and unmethylated WCG sites, respectively.

Currently, DNA demethylation agents, also known as DNMT inhibitors, such as azacitidine (5‐Aza) and decitabine (DEC), exert their therapeutic effects by interfering with the DNA methylation machinery.^48^ These drugs have shown significant efficacy in treating myelodysplastic syndromes,^49^ acute myeloid leukemia,^50^ and other malignancies that are refractory to conventional chemotherapy.^51^ To investigate the effects of these agents, we treated A549 cells with 5‐Aza or DEC for 1, 3 and 5 days and evaluated the changes in DNA methylation and chromatin accessibility using SpliCOOL‐seq (Figure 3A). The global methylation level exhibited a gradual decline with extended treatment. By day 5, the averaged global DNA methylation level decreased from 48.03% to 32.94% in the 5‐Aza group and to 25.13% in the DEC group (Figure 3B). Meanwhile, the DNA methylation patterns also changed dramatically upon both type of DNMT inhibitor treatment. However, the landscape of chromatin accessibility did not show corresponding changes (Figure S3A). Thus, the reduction in methylation levels induced by these hypomethylating agents did not elicit corresponding alterations in chromatin status in vitro.^22^ We observed a marked difference in the demethylation rhythms between the two agents. 5‐Aza induced a gradual decrease in methylation levels over time, both genome‐wide and in gene regions, while DEC treatment did not result in significant changes from day3 to day5 (Figure 3B–D). Similar demethylation patterns were observed across other genomic elements (Figure S3B,C). To further investigate the similarities and differences in the demethylation processes induced by the two agents, we analyzed differential methylation sites (DMSs). 5‐Aza induced more DMSs than DEC, but the magnitude of demethylation changes was smaller in the early stages (Figure S3D), indicating that 5‐Aza results in greater heterogeneity across cells. Although both groups exhibited tens of thousands of hypomethylated DMSs between adjacent stages, there were also thousands of hypermethylated DMSs (Figure S3D). Notably, from day 3 to day 5 in the DEC group, the number and magnitude of hypermethylated and hypomethylated DMSs were nearly equal, resulting in globally unchanged methylation levels (Figure 3D; Figure S3D). In both groups, the hypomethylated DMSs were specific at each time point, suggesting that the demethylation process occurred sequentially for all the target sites (Figure S3E,F).

We also performed clustering of DMRs in each group, which could be divided into 6 clusters based on methylation changes over time (Figure 3E,F; Table S3). Consistent with the DMS analysis, the majority of DMRs underwent demethylation (C4–C6 clusters), while a subset of DMRs transiently gained methylation (C1–C3 clusters). It is well established that during tumorigenesis, numerous tumour suppressor genes (TSGs) are silenced due to DNA hypermethylation. Consequently, one of the antitumour mechanisms of DNMT inhibitors involves the demethylation of CpG islands within gene promoters, thereby reversing the epigenetic silencing and reactivating these TSGs.^52^, ^53^ Specifically, 21 hypermethylated TSGs exhibited demethylation following 5‐Aza treatment, and 17 hypermethylated TSGs showed similar epigenetic changes in response to DEC treatment,^54^ where only five TSGs were commonly demethylated by both inhibitors (Table S4). The methylation level of C4 DMRs dropped to its lowest on the first day of treatment, suggesting these regions might be directly targeted by DNMT. TSGs such as *DAB2IP* and *PAX6* located within these regions may serve as potential biomarkers for early therapeutic response to 5‐Aza and Dec. The C5 and C6 DMRs were later demethylated, indicating they were indirectly regulated by DNMT. Genes associated with these regions—including *LATS1*, *ARL11*, *SELENBP1*, and *DOK1*—may represent candidate biomarkers for sustained treatment efficacy (Figure 3G,H). Furthermore, *KANK1*, which was demethylated in response to both inhibitors (Figure S4A), was significantly downregulated in lung cancer cells and found to enhance drug tolerance. Upregulation of *KANK1* notably suppressed lung cancer cell proliferation, induced apoptosis, and inhibited tumour cell invasion and metastasis.^53^, ^54^, ^55^ These findings support the potential of combining DNMT inhibitors with other therapeutic agents to achieve improved treatment outcomes.

There were also transiently hypermethylated DMRs under both 5‐Aza and DEC treatment, and the regions were enriched in common motifs including GATA1/12/14, LEP, ZNF189 and BPC1 (Figure 3E,F). Further gene ontology (GO) analysis of these DMR‐related genes revealed that both 5‐Aza and DEC induced similar gene regulatory programs. For instance, the demethylated genes (C4–C6) were enriched in developmental‐related processes, cilium assembly and response to stimulus; while the transiently methylated genes were related to G protein‐coupled receptor activity and nervous system processes (Figure S4B). Meanwhile, there were also specific changes in response to the two DMNT inhibitors. For example, cell proliferation, chromatin remodelling and nucleus organization genes were specifically demethylated in the DEC group, while TGFβ responses were only induced in the 5‐Aza group (Figure S4B). Our findings indicate that although different DNMT inhibitors induce global demethylation in cancer cells, they exhibit distinct preferences for target regions, which should be considered in clinical practice.

### 3.4 SpliCOOL‐seq revealed tumour subclones within the tumour lesion

**FIGURE 4:**
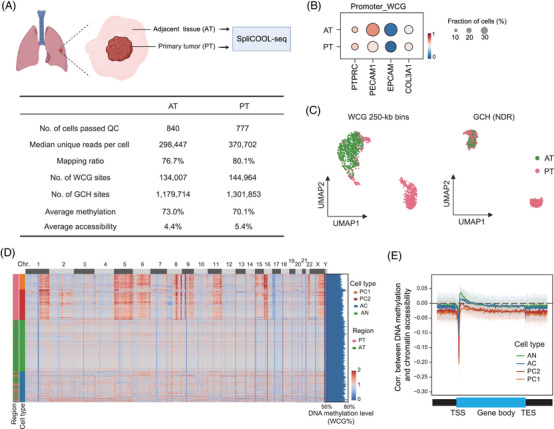
SpliCOOL‐seq revealed tumour subclones within the tumour lesion. (A) Schematic illustration of sampling (upper). The lower table presents the data quality of AT and PT cells in the current experiment. (B) Dot plots showing the promoter WCG methylation levels (−1000 bp to +1000 bp) of representative marker genes in each tissue section. (C) UMAP embedding showing cells based on their DNA methylation levels (WCG in 250‐kb non‐overlapping bins) and chromatin accessibility (GCH based on NDRs). (D) Heatmap showing the high frequency of subchromosome‐scale CNV patterns in PC cells. The bar plot on the right shows the global DNA methylation level of each cell. (E) Spearman correlations between DNA methylation (WCG%) and chromatin accessibility (GCH%) across the gene body in each cell type.

Due to the limitations of current single‐cell methylation sequencing techniques, the genomic and epigenetic heterogeneity of primary cancer cells has been insufficiently characterized. In this study, we applied SpliCOOL‐seq for an integrated analysis of genomic alterations, aberrant DNA methylation and chromatin accessibility in a lung adenocarcinoma (LUAD) tissue sample. The tissue was initially divided into two parts—the primary tumour (PT) and adjacent tissue (AT) (Figure 4A). We obtained a total of 840 cells from the AT, with an average of 298 447 unique reads per cell, and 777 cells from the PT, with an average of 370 702 unique reads per cell (Figure 4A; Figure S4A). Notably, because we extracted the nuclei from the tissues for SpliCOOL‐seq (see methods) and the inherent strategy involving multiple rounds of labelling, the majority of the cells analyzed from both AT and PT were epithelial cells, which exhibited specific hypomethylation and chromatin accessibility in the marker gene *EPCAM* (Figure 4B; Figure S5B). All single cells could be clustered into two groups based on both DNA methylation levels (using WCG 250‐kp bins) and chromatin accessibility (based on NDRs) (Figure 4C). The two cell groups were quite matched in both modalities, demonstrating region specificity, although a few PT cells clustered with those from the AT (Figure 4C; Figure S5C). Further in‐depth examination of somatic CNVs^22^ revealed that all cells could be categorized into four types^47^: primary cancer cells, which were further subdivided into tumour subclone PC1 and PC2, exhibiting a high abundance of somatic CNVs; adjacent normal (AN) cells, which displayed a normal diplotype; and adjacent cancerous (AC) cells, which harbored a small number of somatic CNVs (Figure 4D). Interestingly, the AC cells were dispersed across both AT and PT regions and exhibited DNA methylation and chromatin accessibility patterns more similar to AN cells (Figure S5C), suggesting that these AC cells may represent early cancerous cells.

We further analyzed the changes in the three clones of cancerous cells compared with AN cells. The global methylation levels of the two PC clones were significantly decreased (averagely 68.2% for PC1 and 70.1% for PC2) compared with that of AN cells (73%), while the methylation levels of AC cells remained relatively unchanged (72.6%, Figure S5D,E). Moreover, the interaction between DNA methylation and chromatin accessibility was maintained in AC cells, whereas the PC cells showed a stronger negative correlation between these two modalities (Figure 4E). Together, we captured a group of early cancerous cells that display genomic abnormalities without significant changes in their epigenome.

### 3.5 SpliCOOL‐seq enables the identification of candidate tumour diagnostic targets

We leveraged SpliCOOL‐seq to identify methylation aberrant genes (MAGs) specific to tumour subclones, which may serve as potential biomarkers for early detection and prognosis. PC1 and PC2 subclones exhibited extensive MAGs, with over 60% of MAGs shared between them (Figure S6A; Table S5). In contrast, only 422 MAGs were observed in AC cells, and less than 20% shared with the PC cells, indicating that AC cells may be independently derived. GO analysis demonstrated enrichment of multiple catabolic processes in AC cells and sensory perception of smell in PC cells (Figure S6B), which has been reported to be closely related to tumorigenesis.^55^, ^56^, ^57^ Meanwhile, the hypermethylated genes in AC and PC cells are both enriched in translation repression (Figure S6B), suggesting that the translation regulation of tumour cells is abnormal.^58^, ^59^, ^60^

**FIGURE 5:**
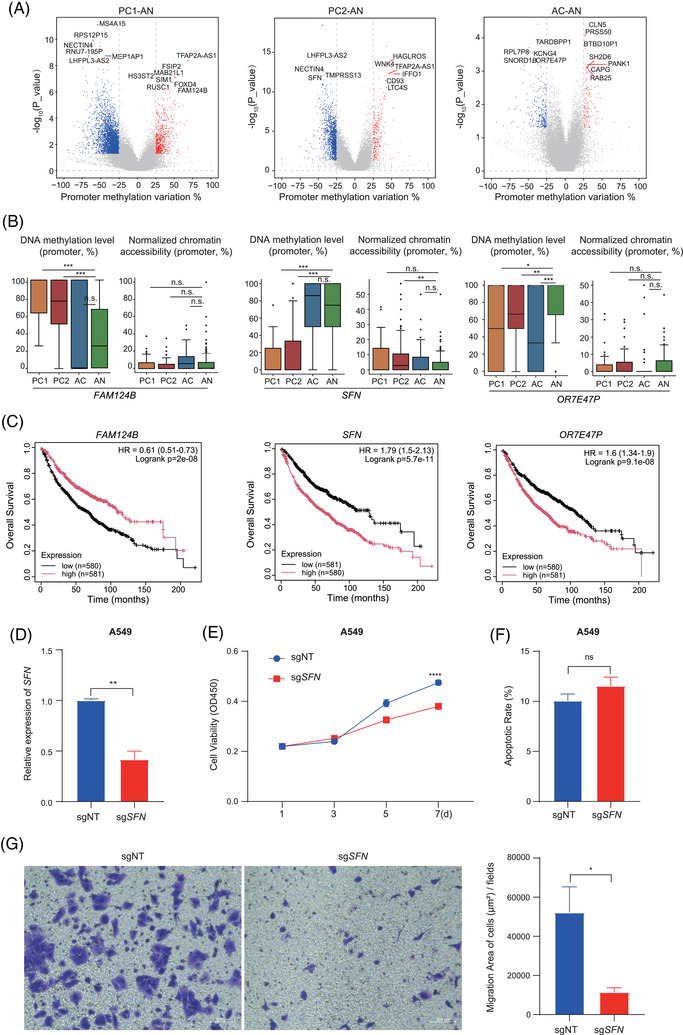
SpliCOOL‐seq enables identification of candidate tumour diagnostic targets. (A) Gene promoter methylation variations between each cancerous clone and AN cells. Top representative genes within each clone are labelled. (B) DNA methylation levels and chromatin accessibility in the promoter regions of the candidate biomarkers across each cell type. The statistical test was carried out using the Wilcoxon rank‐sum test. n.s., no significance; *p < .05; **p < .01; ***p < .001. (C) Overall survival of LUAD patients grouped by the expression of the representative genes. (D) qRT‐PCR detected the expression level of *SFN* in A549 cell lines. The results were presented as the mean ± standard deviation (SD) from three independent experiments. ∗∗*p* < .01. (E) CCK8 assays reveal cell growth curves of A549 with sg*SFN* and sgNT. (F) Cell apoptosis detection assay with indicated cells. Error bars represent the mean ± SD of three independent experiments. ns means no significance. (G) The transwell experiment detects the invasion level of A549 cells.

The top differentially methylated genes identified across the three cancerous clones were highlighted in Figure 5A and may serve as potential DNA methylation biomarkers for LUAD. For example, *HS3ST2* and *LTC4S*, both previously reported in non‐small cell lung cancer (NSCLC), also exhibited significantly hypermethylated in the two PC clones, and higher expression of these genes was associated with improved overall survival,^61^, ^62^ Additionally, we identified the hypermethylated gene *FAM124B* in PC1 cells, the higher expression of which was associated with improved overall survival in LUAD (Figure 5C). This gene has been reported to be hypermethylated in ER+/PR+ breast cancer^63^ and embryonal carcinoma, which were significantly associated with relapse‐free survival.^64^ *SFN* encodes a cell cycle checkpoint protein that binds to translation initiation factors and functions as a regulator of mitotic translation. Research on hepatocellular carcinoma (HCC) has demonstrated that *SFN* promotes the malignant progression of HCC cells and is significantly correlated with a poorer prognosis for HCC patients.^65^ Elevated levels of the *SFN* protein have also been detected in lung tissues, bronchoalveolar lavage fluid, and serum from patients with diffuse alveolar damage.^66^, ^67^ Although it was well known that *SFN* is highly expressed in LUAD and promotes early adenocarcinoma progression,^68^ the regulatory mechanism underlying the aberrant expression of this gene remained unclear. Our results indicated that the *SFN* promoter region exhibits significantly lower methylation levels and higher chromatin accessibility in PC2 cells, while low *SFN* expression correlates with better prognosis in LUAD (Figure 5B,C). This indicated that *SFN* facilitates LUAD progression via a mechanism involving promoter hypomethylation and enhanced chromatin accessibility, leading to its transcriptional upregulation and stimulation of cell proliferation. The AC cell‐specific hypomethylated gene *OR7E47P* significantly affected the overall survival in LUAD patients (Figure 5A–C), further supporting it as an early diagnostic marker.^69^, ^70^ These findings highlight the potential of SpliCOOL‐seq in uncovering novel DNA methylation biomarkers with clinical relevance.

We then validated the biological role of *SFN* in A549 and H460 cells by knocking out the gene with CRISPR (sg*SFN*). The expression of SFN was significantly downregulated according to qRT‐PCR (Figure 5D; Figure S6C). Functional assays demonstrated that *SFN* silencing significantly inhibited cell proliferation and migration, but showed no significant effect on apoptosis (Figure 5E–G; Figure S6D,E). Further RNA‐seq analysis revealed that 216 genes were downregulated in the sg*SFN* cells (Figure S5F,G; Table S8). Among these genes, we identified *FGF2*, which can bind to FGFR and subsequently activate pathways such as Ras‑MAPK and PI3K, thereby promoting cell proliferation, angiogenesis, cancer progression, and poor prognosis.^71^ Additionally, sg*SFN* could decrease cell motility via the MAP4K4‑associated migration pathway^72^ (Figure S6G). GO enrichment of the downregulated genes also highlighted terms related to cell proliferation and migration (Figure S6H).

### 3.6 Cancer cells exhibit accelerated epigenetic ageing

**FIGURE 6:**
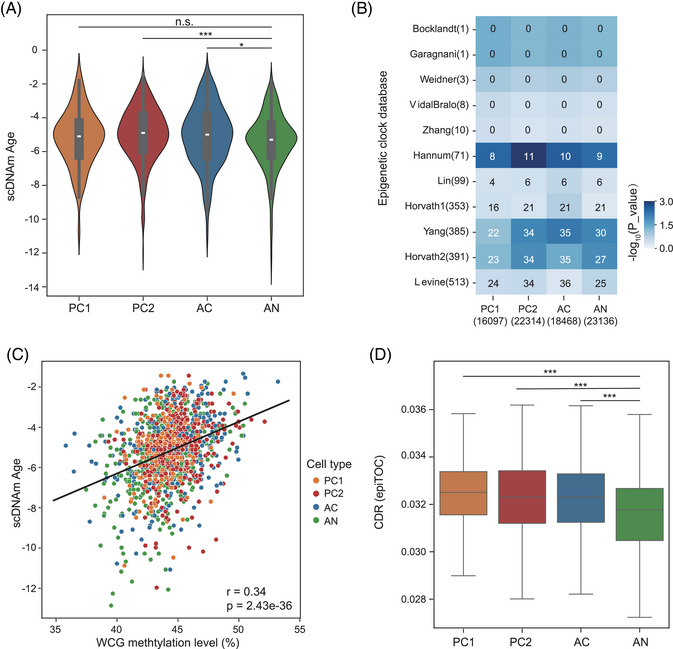
Cancer cells exhibit accelerated ageing. (A) Violin plots depicting the scDNAm Age distribution in each clone. (B) Heatmap showing the intersection of the top 10% age‐associated CG sites identified in each clone and those identified in different epigenetic clocks. The number of CG sites is presented within the respective brackets. (C) Dot plot illustrating the correlation between global methylation levels of the predictive CG sites and scDNAm Age for each cell, with point colours representing distinct clones. (D) Box plots depicting the cell division rate distribution in each clone.

The development of epigenetic clocks to predict physiological age based on DNA methylation has become a pivotal resource for comparative epigenomic studies, shedding light on the mechanisms underlying epigenetic ageing.^73^, ^74^, ^75^, ^76^ We sought to determine whether SpliCOOL‐seq could capture changes in ageing status during tumorigenesis. To this end, we adopted the scAge^32^ framework to calculate the epigenetic age of individual cells. First, we utilized the 450k methylation array data of LUAD from The Cancer Genome Atlas (TCGA)^77^ for model training, which generated a ranked list of CG sites correlated with age. Top 10% ageing‐associated sites were extracted to predict the DNA methylation (DNAm) age. On average, 1823 WCG sites were detected in each cell overlapped with the TCGA array data. To validate the accuracy of the model, we subset a cohort from the TCGA dataset and randomly downsampled the detected CG sites to 2300 per sample to simulate the sparse features of single‐cell data. The predicted DNAm ages were positively correlated with the chronological ages of the samples (Figure S6A). Based on the in‐home trained model, we found that all three cancerous clones showed increased epigenetic age compared with the AN cells (Figure 6A), consistent with previous findings that cancer tissues exhibit significant age acceleration.^78^, ^79^, ^80^ Then we extracted the DNAm age‐related CG sites in each cluster of cells and compared these sites with the CpG markers reported in existing epigenetic clocks,^78^, ^79^, ^80^, ^81^, ^82^, ^83^, ^84^, ^85^, ^86^, ^87^, ^88^ finding they were significantly overlapped with the markers in three canonical DNA methylation‐based epigenetic clocks—Hannum,^79^ Yang^80^ and Horvath2^87^ (Figure 6B). We employed CpG‐transformer^33^ to impute missing values in our single‐cell DNA methylation (scDNAm) data, specifically targeting unknown methylation levels at WCG sites shared with array data, and leveraged three epigenetic clocks to predict methylation age at single‐cell resolution. Notably, the Horvath2 clock demonstrated concordant results with our model predictions (Figure S6B). This convergence underscores the evolutionary conservation of epigenetic regulatory frameworks and validates the predictive accuracy of our model. The relationship between the methylation levels of the predictive CG sites and the scDNAm age revealed a positive correlation across all cell clones (Figure 6C), suggesting that the majority of the ageing‐associated sites gained methylation over time, which was even more intense in LUAD cancer cells. Further division of the predictive sites based on their correlation with age showed that 80.3% sites showed age‐positive correlation in both tumour and normal cell clones (Figure S6C). These findings indicate that ageing and LUAD tumorigenesis may share similar epigenetic signals, a phenomenon also noted in other types of cancer.^89^, ^90^, ^91^

The risk of tumorigenesis is closely associated with the cumulative number of cell divisions occurring within the pool of potential stem cells in a given tissue.^92^, ^93^ Mitotic clock models based on DNA methylation changes had garnered significant attention, as they were specifically designed to track DNA methylation abnormalities accumulated during cell divisions.^94^, ^95^, ^96^ The epigenetic mitotic clock has been shown to exhibit increased age acceleration in both cancerous and precancerous lesions, suggesting its potential utility as a predictive tool for assessing cancer risk.^94^, ^97^, ^98^, ^99^ We therefore utilized the epiTOC model^94^ to estimate the cell division rate (CDR). The result revealed significantly elevated cell division rates in PC and AC subclones compared with AN cells (Figure 6D), supporting the link between mitotic activity and epigenetic ageing.^93^ To specify how tumorigenesis affects epigenetic ageing, we extracted sites positively correlated with age that also showed elevated methylation levels in tumour cells. We identified a total of 124 sites from all three cancerous clones, which corresponded to 138 genes (Table S6). GO analysis of these genes showed significant enrichment in calcium ion homeostasis, organ morphogenesis and neuronal system (Figure S6D), which have been previously reported to be associated with cellular senescence or tumorigenesis.^100^, ^101^, ^102^, ^103^, ^104^ Specifically, *VSIG4* identified in PC1 cells has been identified as a biomarker for adipose tissue macrophages in aged mice. Meanwhile, its expression was markedly induced in the tumour‐adjacent stroma in both spontaneous and xenograft models of lung cancer.^105^ *IL17C* and *CCL11* (eotaxin) identified in PC2 cells exhibited a significant correlation with age, tumour stage, metastasis, lesion count, and tumour burden.^106^, ^107^ *NAF1* identified in AC cells has been reported to modulate calcium ion concentration, reactive oxygen species (ROS) levels, and iron metabolism signalling pathways to fulfil its regulatory functions.^108^ Together, we identified a series of abnormal DNA methylation regulations that are shared in ageing and tumorigenesis.

## 4 DISCUSSION

SpliCOOL‐seq represents a significant advancement in single‐cell multi‐omics profiling, which exhibits superior read quality and sensitivity, enabling precise discrimination among tumour cells based on genomic and multiple epigenetic characteristics. We employed a combination of 48 × 96 ligation barcodes along with indexed PCR amplification to label individual cells. After the ligation steps, each tube can be allocated 200 cells to control the proportion of cell doublets in a quite low ratio (less than 5%). For each experiment, the recovered cell number can reach around 20 000 when using 96 PCR indexes. This approach offers flexibility for application across various cell throughput requirements. Of course, SpliCOOL‐seq does present certain limitations. First, in situ methylation of the nucleus exhibits lower efficiency compared with the methylation of naked DNA^8^ and is less stable than bisulfite treatment, which may introduce bias in the baseline of GCH methylation. This way, it is unreliable for the absolute quantification of the global accessibility of the chromatin in individual cells. Second, although the crosslinking and sodium dodecyl sulfate (SDS)‐based nucleosome depletion treatment effectively disrupts nucleosomes (Figure S1F), it can also cause significant damage to the nuclear structure, even in cross‐linked nuclei. Consequently, the method demands a large starting population of cells—on the order of millions—and results in a cell loss rate of approximately 75%. This substantial attrition particularly affects fragile cell types, leading to potential cell type bias in the final analysis. Third, given that the endogenous CH methylation is well‐characterized in the nervous system, GCH methylation may serve as an imprecise indicator of chromatin accessibility when applied in these tissue contexts. Consequently, this technique may have limited applicability in neurological research.

DNA methylation biomarkers demonstrate significant potential and distinct advantages over conventional cancer biomarkers (such as cancer‐associated genes and proteins) for early cancer screening, non‐invasive diagnosis, tumour subtyping, prognostic assessment, therapeutic response monitoring, and recurrence prediction. These advantages stem from their high tissue specificity, occurrence as early molecular events in carcinogenesis, superior performance in liquid biopsies (offering stability and tumour origin traceability), digital signal nature, and capacity for multiplexed detection.^109^, ^110^, ^111^, ^112^ Consequently, DNA methylation biomarkers hold considerable promise to surpass or effectively complement traditional genetic mutation and protein biomarkers. Although it is commonly inferred that DNA methylation contributes to gene silencing, this remains a statistical association, and the causal relationship—whether methylation drives silencing or is a consequence of it—remains unclear. By integrating chromatin accessibility analysis, we can determine whether the hypermethylated region coincides with a significant reduction in chromatin accessibility (i.e., a transition from an “open” to a “closed” chromatin state), thereby directly linking DNA methylation to a key structural change in chromatin and providing mechanistic insight into gene silencing. Furthermore, using DNA methylation data alone, it is challenging to definitively interpret whether increased gene body methylation promotes or represses transcription. When combined with chromatin accessibility profiling, it should better support the interpretation of a gene in a suppressed or actively transcribed state. Therefore, our method has identified several novel DNA methylation biomarkers in LUAD, where *FAM124B* and *SFN* may serve as potential therapeutic targets for the later stages of tumorigenesis. Furthermore, SpliCOOL‐seq uncovered adjacent cancerous cells within the LUAD tissue, which harboured somatic CNVs but retained near‐normal DNA methylation levels. These AC cells, dispersed across the tumour and adjacent regions, may represent early malignant transformations preceding epigenomic dysregulation. This finding underscores the importance of integrating genomic and epigenomic data to capture incipient tumorigenesis. Moreover, the hypomethylated gene *OR7E47P* in AC cells correlated with poor survival in LUAD. It is worth examining whether diminishing these pathways prevents tumour development.

In this study, we presented the first report on the heterogeneity of scDNAm age acceleration and mitosis acceleration among tumour subclones as predicted from a single‐cell perspective. While SpliCOOL‐seq revealed accelerated epigenetic ageing in tumour subclones, our scDNAm age model was trained on bulk 450k methylation data from TCGA, which differs fundamentally from SpliCOOL‐seq profiles in coverage and resolution. This discrepancy may introduce systematic biases for the model. Future studies could refine these models using single‐cell‐specific epigenetic clocks trained on matched datasets to mitigate technical variability.

## AUTHOR CONTRIBUTIONS

**Xiao‐Ying Fan**: Writing—review & editing, writing—original draft, supervision, project conceptualization and funding acquisition. **Qing‐Mei Shen**: Writing—review & editing, writing—original draft, validation, methodology, investigation, data curation. **En‐Ze Deng**: Writing—original draft and visualization. **Ling‐Luo**: verification of *SFN* in A549 and H460 cells. **Jing‐Na Zhang**: cultured the cell lines and bulk RNA‐seq library preparation. **Qi‐Feng Yang**: Collected the LUAD tissue sample. **Dan Su**: Investigation. All authors have read the manuscript, offered feedback and approved it before submission.

## CONFLICT OF INTEREST STATEMENT

The authors declare no conflict of interest.

## CODE AVAILABILITY

The code for pre‐processing SpliCOOL‐seq data is accessed online at https://github.com/fanxylab/SpliCOOL‐seq.git.

## ETHICS STATEMENT AND CONSENT TO PARTICIPATE

Approval of the study was obtained from the Institutional Review Board of the First Affiliated Hospital of Guangzhou Medical University (reference number: ES‐2025‐K007). To investigate the genomic and epigenetic heterogeneity of primary cancer cells using SpliCOOL‐seq, we obtained a sample from a patient who had undergone lung resection for pulmonary invasive adenocarcinoma. Written informed consent was provided by the patient before sampling.

## CONSENT FOR PUBLICATION

Not applicable.

## Supporting information



Supporting Information

Supporting Information

Supporting Information

Supporting Information

Supporting Information

Supporting Information

Supporting Information

Supporting Information

Supporting Information

## Data Availability

The raw and processed sequencing data in this study have been deposited in the Genome Sequence Archive in the National Genomics Data Center, Chinese National Center for Bioinformation/ Beijing Institute of Genomics, Chinese Academy of Sciences (GSA‐Human: HRA010465), which are publicly accessible under accession number PRJCA036138.
