# Supplementary material for: High‐throughput single‐cell DNA methylation and chromatin accessibility co‐profiling with SpliCOOL‐seq
Source: Clin Transl Med. 2026 Jan 28;16(2):e70584. doi: 10.1002/ctm2.70584 (PMC12848781; doi:10.1002/ctm2.70584)
Supplement: Supplementary file 9 — Supporting Information [file CTM2-16-e70584-s004.pdf]

Fig. S1

A

Cells fixed con.	Cells recovered from one SpliCOOL-seq experiment	Average No. of CpGs coverage/unique read
0.75 %	< 10 %	0.92
1.5 %	~ 25 %	0.94
3 %	~ 25%	0.82

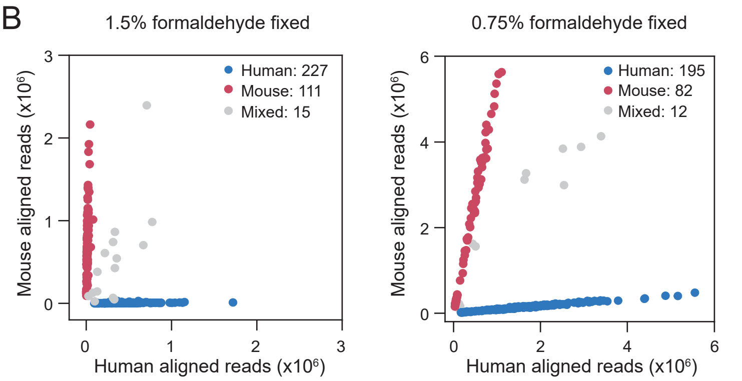

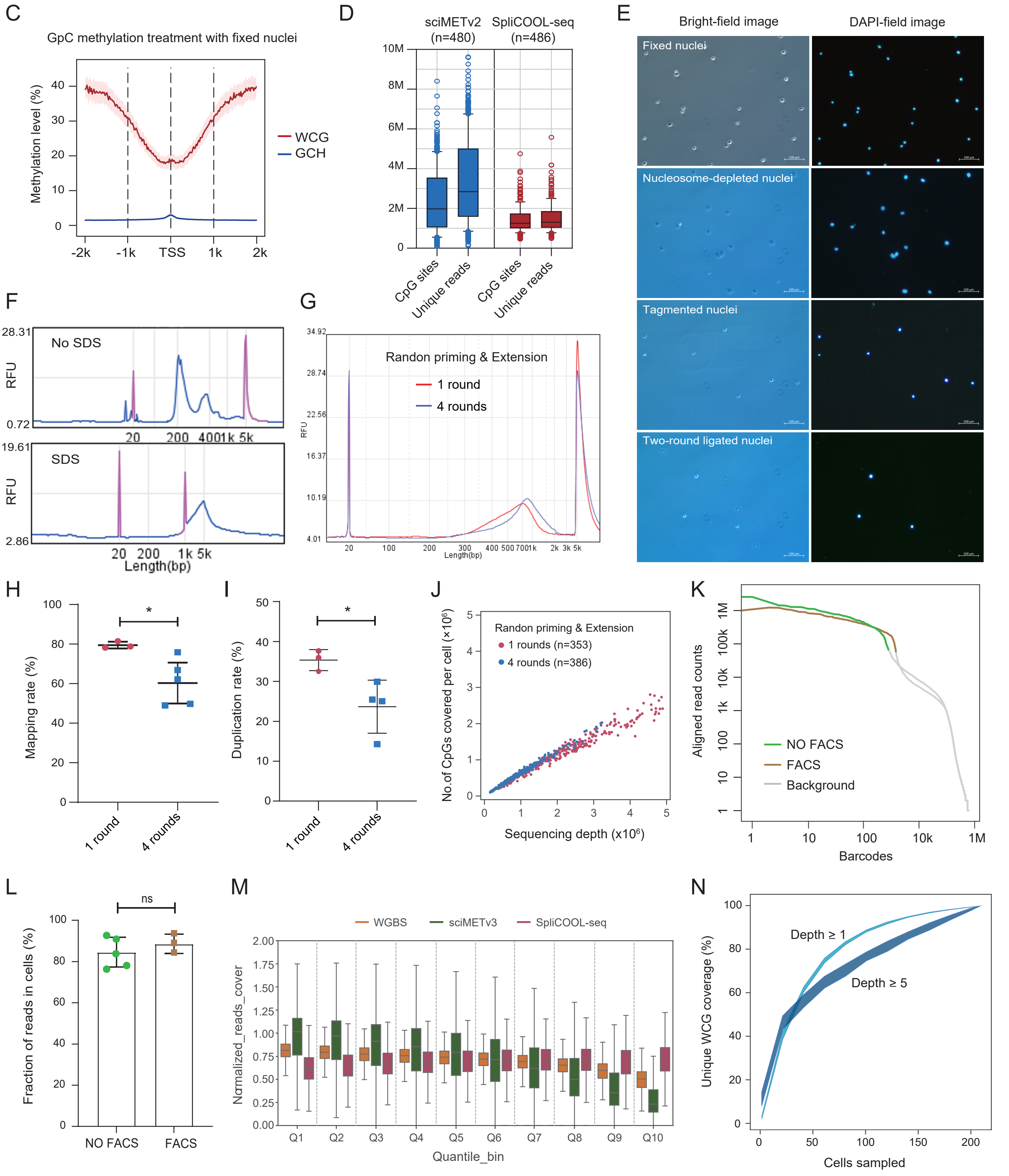

Fig. S2

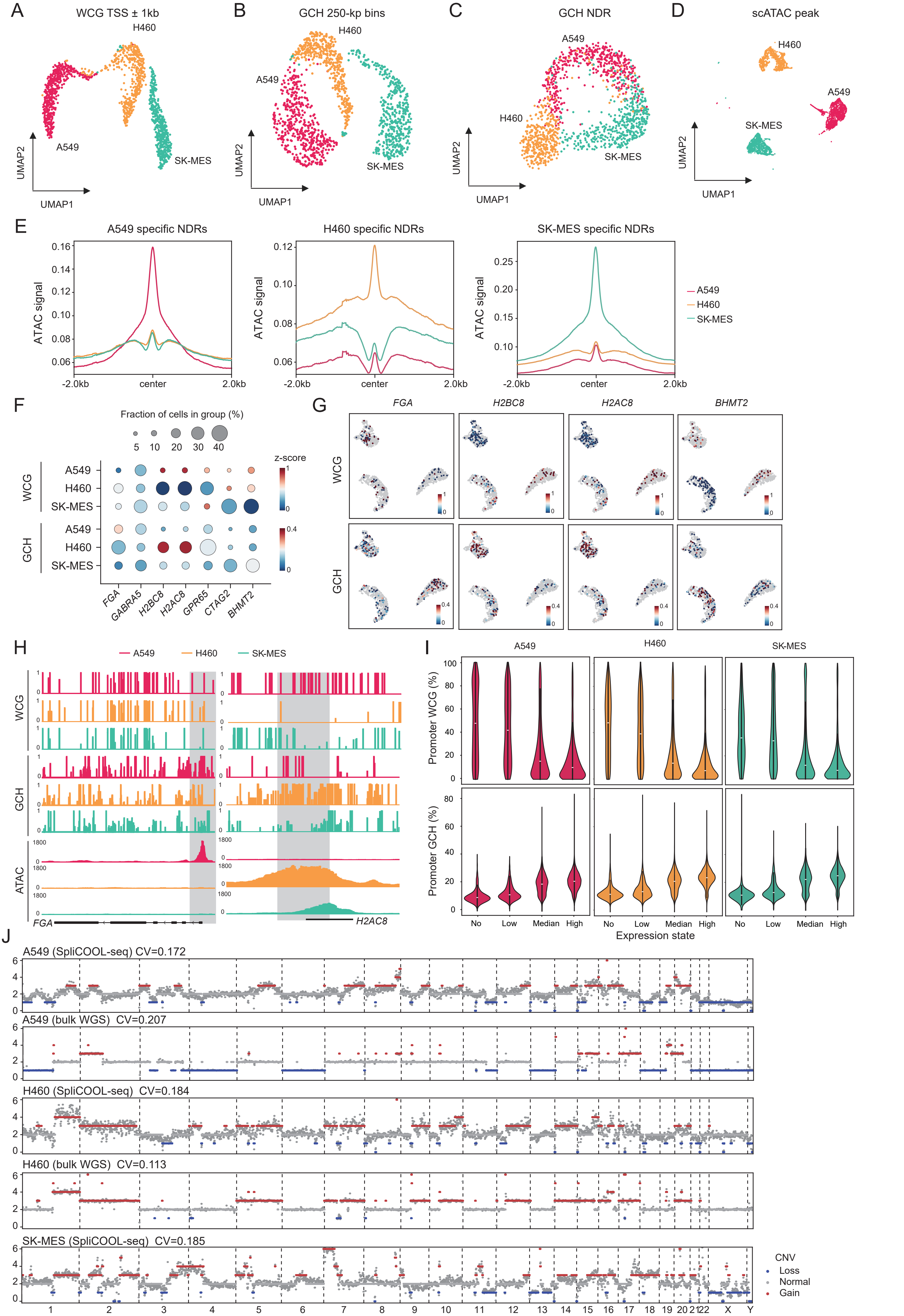
A
WCG TSS ± 1kb
H460
A549
SK-MES
UMAP1
UMAP2
B
GCH 250-kp bins
C
GCH NDR
D
scATAC peak
E
A549 specific NDRs
H460 specific NDRs
SK-MES specific NDRs
ATAC signal
-2.0kb
center
2.0kb
F
Fraction of cells in group (%)
5 10 20 30 40
z-score
WCG
GCH
FGA
GABRA5
H2BC8
H2AC8
GPR65
CTAG2
BHMT2
G
H
ATAC
I
Promoter WCG (%)
Promoter GCH (%)
No
Low
Median
High
Expression state
J
A549 (SpliCOOL-seq) CV=0.172
A549 (bulk WGS) CV=0.207
H460 (SpliCOOL-seq) CV=0.184
H460 (bulk WGS) CV=0.113
SK-MES (SpliCOOL-seq) CV=0.185
CNV
Loss
Normal
Gain
1 2 3 4 5 6 7 8 9 10 11 12 13 14 15 16 17 18 19 20 21 22 X Y

Fig. S3

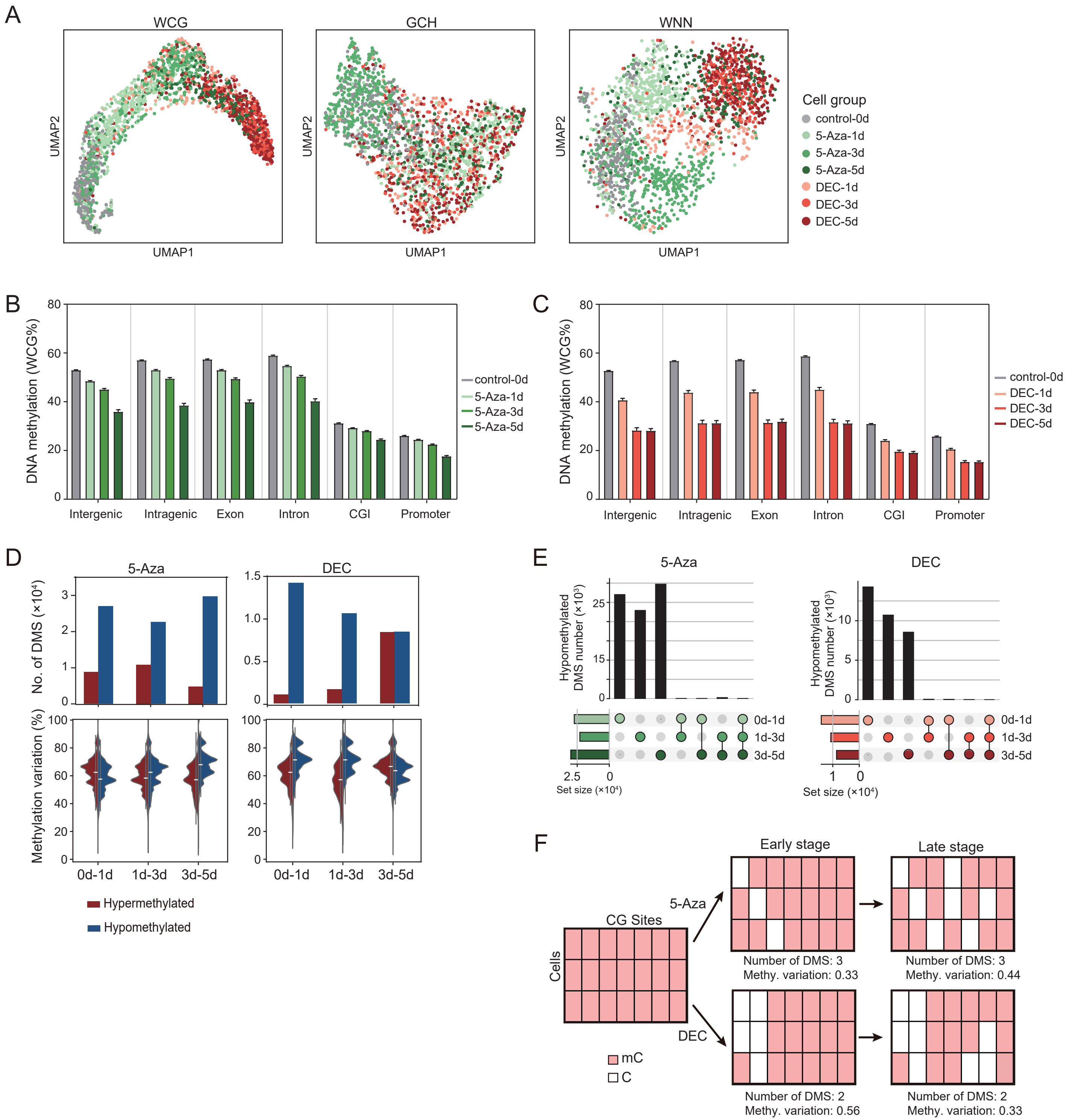

A
WCG
GCH
WNN
UMAP1
UMAP2
Cell group
control-0d
5-Aza-1d
5-Aza-3d
5-Aza-5d
DEC-1d
DEC-3d
DEC-5d
B
C
DNA methylation (WCG%)
Intergenic
Intragenic
Exon
Intron
CGI
Promoter
control-0d
5-Aza-1d
5-Aza-3d
5-Aza-5d
DEC-1d
DEC-3d
DEC-5d
D
5-Aza
DEC
No. of DMS (×10⁴)
Methylation variation (%)
0d-1d
1d-3d
3d-5d
Hypermethylated
Hypomethylated
E
5-Aza
DEC
Hypomethylated DMS number (×10³)
0d-1d
1d-3d
3d-5d
Set size (×10⁴)
F
Early stage
Late stage
CG Sites
Cells
5-Aza
DEC
Number of DMS: 3
Methy. variation: 0.33
Number of DMS: 3
Methy. variation: 0.44
Number of DMS: 2
Methy. variation: 0.56
Number of DMS: 2
Methy. variation: 0.33
mC
C

Fig. S4

A

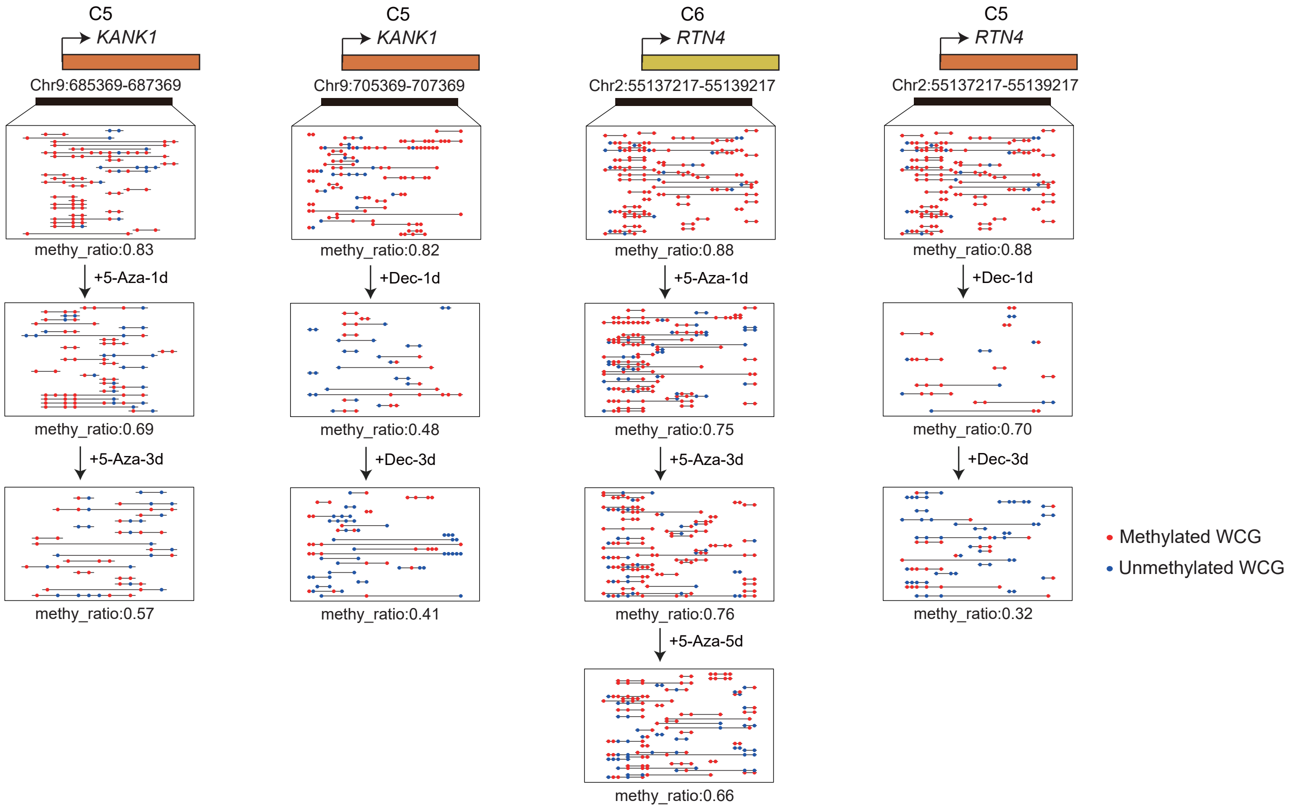

C5
KANK1
Chr9:685369-687369
methy_ratio:0.83
+5-Aza-1d
methy_ratio:0.69
+5-Aza-3d
methy_ratio:0.57
C5
KANK1
Chr9:705369-707369
methy_ratio:0.82
+Dec-1d
methy_ratio:0.48
+Dec-3d
methy_ratio:0.41
C6
RTN4
Chr2:55137217-55139217
methy_ratio:0.88
+5-Aza-1d
methy_ratio:0.75
+5-Aza-3d
methy_ratio:0.76
+5-Aza-5d
methy_ratio:0.66
C5
RTN4
Chr2:55137217-55139217
methy_ratio:0.88
+Dec-1d
methy_ratio:0.70
+Dec-3d
methy_ratio:0.32
Methylated WCG
Unmethylated WCG

B

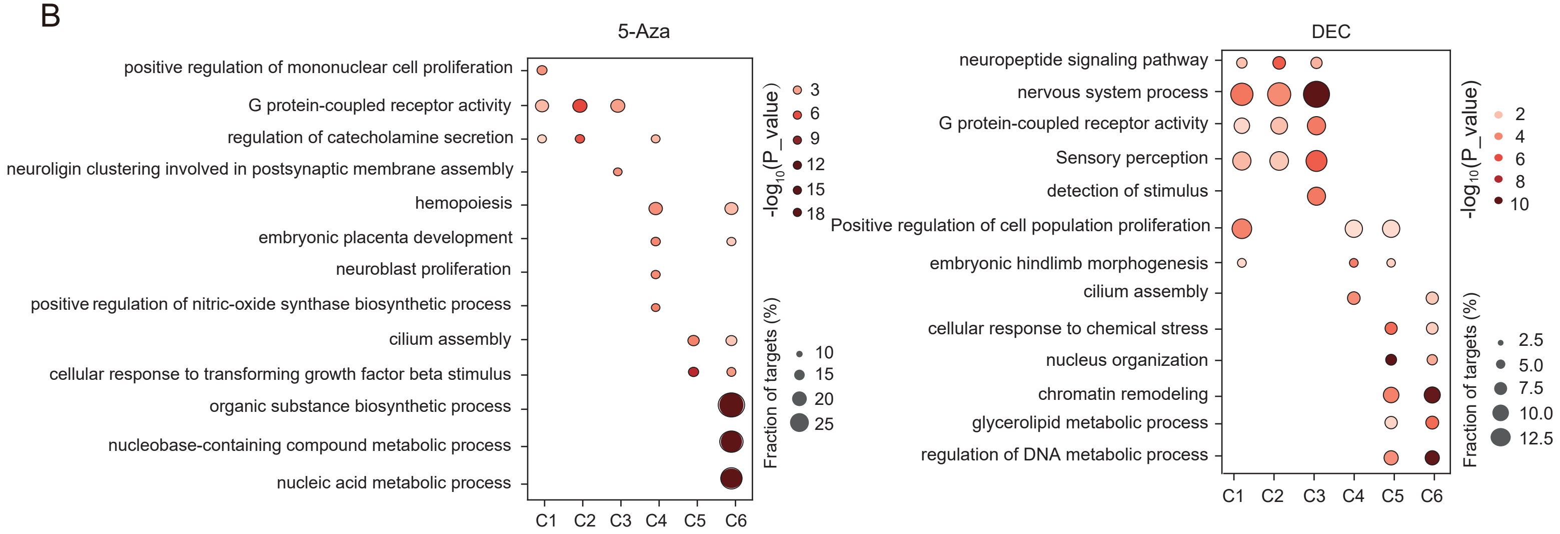

5-Aza
positive regulation of mononuclear cell proliferation
G protein-coupled receptor activity
regulation of catecholamine secretion
neuroligin clustering involved in postsynaptic membrane assembly
hemopoiesis
embryonic placenta development
neuroblast proliferation
positive regulation of nitric-oxide synthase biosynthetic process
cilium assembly
cellular response to transforming growth factor beta stimulus
organic substance biosynthetic process
nucleobase-containing compound metabolic process
nucleic acid metabolic process
C1 C2 C3 C4 C5 C6
-log10(P_value)
3 6 9 12 15 18
Fraction of targets (%)
10 15 20 25
DEC
neuropeptide signaling pathway
nervous system process
G protein-coupled receptor activity
Sensory perception
detection of stimulus
Positive regulation of cell population proliferation
embryonic hindlimb morphogenesis
cilium assembly
cellular response to chemical stress
nucleus organization
chromatin remodeling
glycerolipid metabolic process
regulation of DNA metabolic process
C1 C2 C3 C4 C5 C6
-log10(P_value)
2 4 6 8 10
Fraction of targets (%)
2.5 5.0 7.5 10.0 12.5

Fig. S5

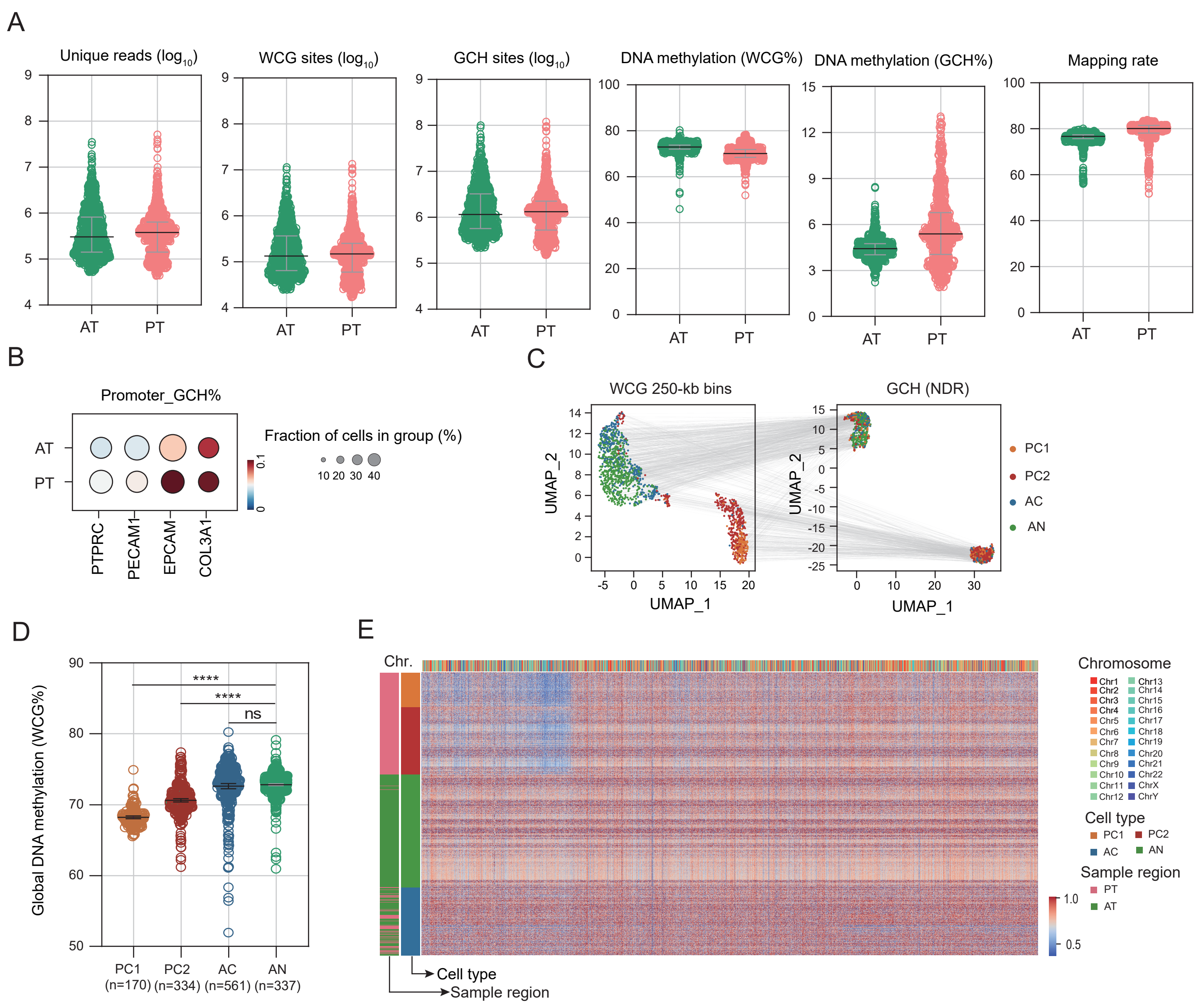
A
Unique reads (log10)
WCG sites (log10)
GCH sites (log10)
DNA methylation (WCG%)
DNA methylation (GCH%)
Mapping rate
AT
PT
B
Promoter_GCH%
Fraction of cells in group (%)
10 20 30 40
PTPRC
PECAM1
EPCAM
COL3A1
C
WCG 250-kb bins
GCH (NDR)
UMAP_1
UMAP_2
PC1
PC2
AC
AN
D
Global DNA methylation (WCG%)
****
****
ns
PC1 (n=170)
PC2 (n=334)
AC (n=561)
AN (n=337)
E
Chr.
Chromosome
Chr1 Chr2 Chr3 Chr4 Chr5 Chr6 Chr7 Chr8 Chr9 Chr10 Chr11 Chr12 Chr13 Chr14 Chr15 Chr16 Chr17 Chr18 Chr19 Chr20 Chr21 Chr22 ChrX ChrY
Cell type
PC1 PC2 AC AN
Sample region
PT
AT
1.0
0.5
Cell type
Sample region

Fig. S6

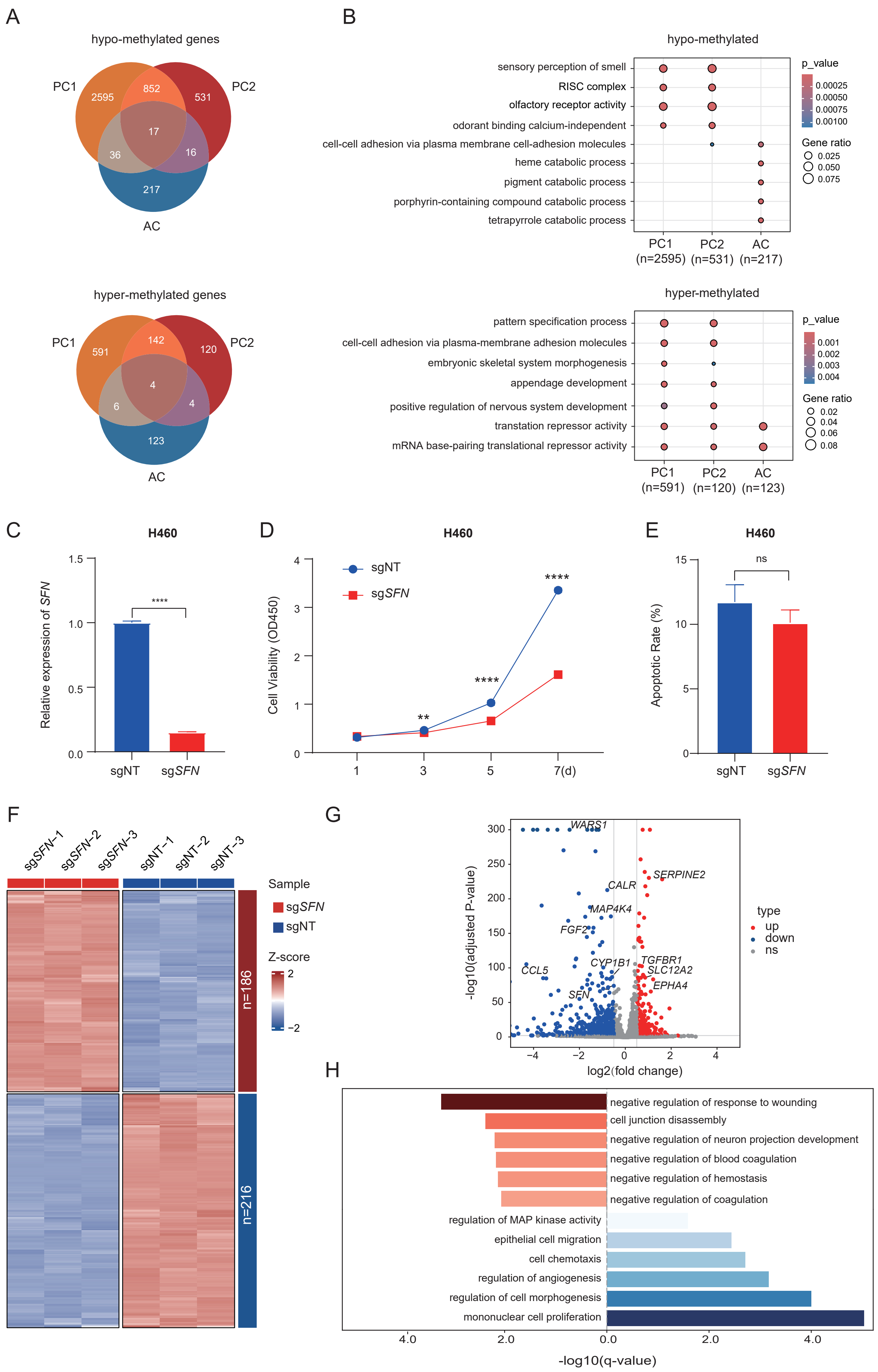

A
hypo-methylated genes
PC1
PC2
AC
2595
852
531
17
36
16
217
hyper-methylated genes
591
142
120
4
6
4
123
B
hypo-methylated
sensory perception of smell
RISC complex
olfactory receptor activity
odorant binding calcium-independent
cell-cell adhesion via plasma membrane cell-adhesion molecules
heme catabolic process
pigment catabolic process
porphyrin-containing compound catabolic process
tetrapyrrole catabolic process
PC1 (n=2595)
PC2 (n=531)
AC (n=217)
p_value
0.00025
0.00050
0.00075
0.00100
Gene ratio
0.025
0.050
0.075
hyper-methylated
pattern specification process
cell-cell adhesion via plasma-membrane adhesion molecules
embryonic skeletal system morphogenesis
appendage development
positive regulation of nervous system development
transtation repressor activity
mRNA base-pairing translational repressor activity
PC1 (n=591)
PC2 (n=120)
AC (n=123)
p_value
0.001
0.002
0.003
0.004
Gene ratio
0.02
0.04
0.06
0.08
C
H460
Relative expression of SFN
1.5
1.0
0.5
0.0
****
sgNT
sgSFN
D
H460
Cell Viability (OD450)
sgNT
sgSFN
**
****
****
4
3
2
1
0
1
3
5
7(d)
E
H460
Apoptotic Rate (%)
ns
15
10
5
0
sgNT
sgSFN
F
sgSFN-1
sgSFN-2
sgSFN-3
sgNT-1
sgNT-2
sgNT-3
Sample
sgSFN
sgNT
Z-score
2
−2
n=186
n=216
G
-log10(adjusted P-value)
300
250
200
150
100
50
0
−4
−2
0
2
4
log2(fold change)
WARS1
SERPINE2
CALR
MAP4K4
FGF2
CCL5
CYP1B1
TGFBR1
SLC12A2
EPHA4
SFN
type
up
down
ns
H
negative regulation of response to wounding
cell junction disassembly
negative regulation of neuron projection development
negative regulation of blood coagulation
negative regulation of hemostasis
negative regulation of coagulation
regulation of MAP kinase activity
epithelial cell migration
cell chemotaxis
regulation of angiogenesis
regulation of cell morphogenesis
mononuclear cell proliferation
4.0
2.0
0.0
2.0
4.0
-log10(q-value)

Fig. S7

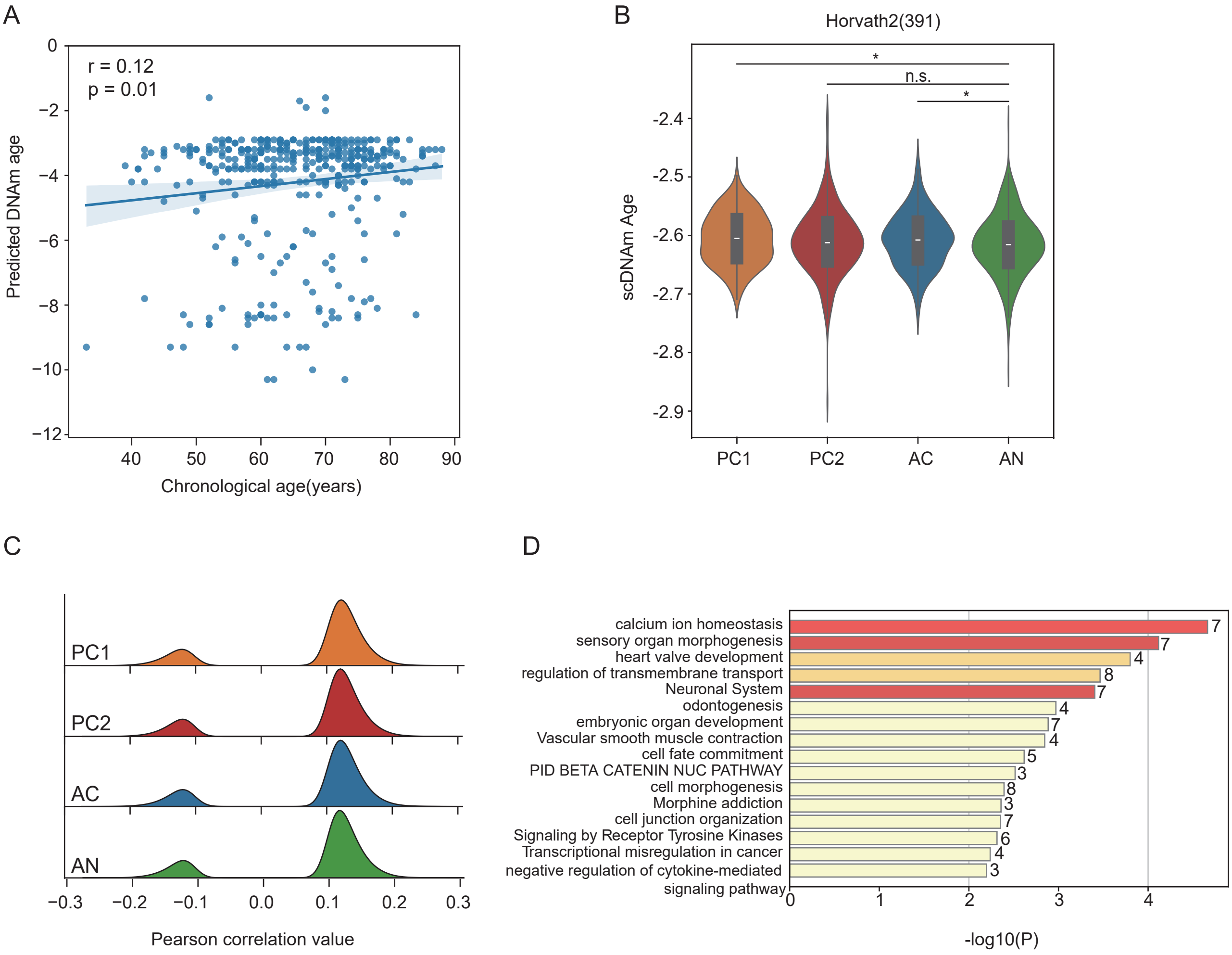

A
r = 0.12
p = 0.01
Predicted DNAm age
Chronological age(years)
B
Horvath2(391)
scDNAm Age
*
n.s.
*
PC1
PC2
AC
AN
C
PC1
PC2
AC
AN
Pearson correlation value
D
calcium ion homeostasis
sensory organ morphogenesis
heart valve development
regulation of transmembrane transport
Neuronal System
odontogenesis
embryonic organ development
Vascular smooth muscle contraction
cell fate commitment
PID BETA CATENIN NUC PATHWAY
cell morphogenesis
Morphine addiction
cell junction organization
Signaling by Receptor Tyrosine Kinases
Transcriptional misregulation in cancer
negative regulation of cytokine-mediated signaling pathway
-log10(P)
